# Infant presence and mother-infant conflict predict fetal loss in wild mandrills

**DOI:** 10.1371/journal.pbio.3003970

**Published:** 2026-11-03

**Authors:** Nikolaos Smit, Berta Roura-Torres, Axelle Delaunay, Alice Baniel, Elise Huchard, Marie J. E. Charpentier

**Affiliations:** 1 Department of Primate Behavior and Evolution, Max Planck Institute for Evolutionary Anthropology, Leipzig, Germany; 2 Department of Biology, University of Turku, Turku, Finland; 3 Institute of Evolutionary Sciences of Montpellier (ISEM), University of Montpellier, CNRS, IRD, EPHE, Montpellier, France; 4 Behavioral Ecology and Sociobiology Unit, German Primate Center, Leibniz Institute for Primate Research, Göttingen, Germany; 5 Department of Sociobiology/Anthropology, Johann-Friedrich-Blumenbach, Institute of Zoology and Anthropology, Georg-August University Göttingen, Göttingen, Germany; The Australian National University, AUSTRALIA

## Abstract

Female fitness depends on reproductive pace and offspring survival, both of which are determined by the ability to balance resource allocation between current and future reproduction. Using 11 years of data on 356 pregnancies (52 of which resulted in fetal loss) from 117 wild female mandrills, we show that females who conceive while still caring for a young infant experience a greater probability of fetal loss as well as increased mother-infant behavioral conflict in the form of maternal rejection. In addition, females whose infants throw more tantrums show a greater probability of fetal loss, consistent with observations from other species where living offspring can interfere in parental reproduction. Finally, females who lose a fetus show greater subsequent reproductive success (produce more surviving infants and show shorter inter-birth intervals) than females who give birth but their infants die soon after birth. These findings suggest that fetal loss may represent an adaptive physiological response to the energetic constraints imposed by overlapping gestation and maternal care, potentially limiting the fitness costs associated with short inter-birth intervals and concurrent investment in multiple dependent offspring.

## Abstract 2

Maternal fitness depends on balancing investment between current and future reproduction. This study shows that, in wild mandrills, infant presence and tantrum behavior predict fetal loss, consistent with an adaptive response that reduces the costs of concurrent reproductive investment.

## Introduction

Life-history theory suggests that organisms cannot maximize both offspring quantity and quality, as producing more offspring inevitably dilutes the investment allocated to each [1–3]. Indeed, large clutch and litter sizes or short inter-birth intervals often compromise offspring growth rate and survival [4–11]. In female mammals, reproducing at a faster pace without compromising offspring quality likely depends on the capacity to restore the energetic reserves depleted by the previous reproductive event and, when offspring care overlaps with a new pregnancy, to sustain the energetic demands of both [12–16]. In addition, simultaneous pregnancy and maternal care may result in ‘sibling competition’ between the living offspring and the fetus over maternal resource allocation (e.g., nutrient transfer [17,18]). Therefore, a female who conceives soon after giving birth faces concurrent allocation demands from both infant and fetus, giving rise to interacting physiological and behavioral conflicts among the female, her living infant, and her fetus, which may influence the survival of both offspring [10,19]. These conflicts may be particularly intensified when conception occurs before weaning of the living infant, as mothers reduce investment while infants continue to solicit high levels of care.

In this study, we test whether females who conceive while still caring for an unweaned offspring experience a greater risk of fetal loss, reflecting constraints limiting female reproductive pace. Such a pattern is expected when female having already invested substantially into their current, living offspring, prioritize these offspring over fetal survival. Using 11 years of detailed life-history, behavioral, and reproductive data from a large wild population of mandrills (*Mandrillus sphinx*), we first examine whether the probability of fetal loss is linked to (i) a short temporal proximity to the previous pregnancy, which may leave females physiologically or morphologically constrained [14,15] or (ii) the concurrent energetic demands of infant care, which may limit the energy available to invest in fetal development. Second, we test whether pregnant females, compared to nonpregnant females, provide less maternal care to their living infants and experience increased mother-infant behavioral conflict, as they face additional energetic constraints while their infants still need and demand high levels of maternal care. Third, we test whether greater maternal care and/or mother-infant behavioral conflict increase the probability of fetal loss. Finally, we test whether fetal loss mitigates the costs of short inter-birth intervals, such as increased infant mortality—a mechanism that may operate when females cannot sustain investment in two closely conceived offspring and prioritize the living infant which embodies greater overall investment.

In mandrills, reproduction is seasonal, with alternating mating and birth periods of roughly 6 months [20]. Females typically conceive every other mating season but some females—often high-ranking ones—conceive in consecutive mating seasons while still caring for unweaned infants (median inter-birth interval: 566 days, range 323–1,024; [20]). This natural variation in inter-birth interval length provides a valuable framework for investigating the costs of accelerated reproduction. In addition, female mandrills display conspicuous sexual and pregnancy swellings (Fig 1) which, combined with detailed monitoring of reproductive patterns, allow reliable identification of pregnancies and detection of both live births and fetal losses. During this study, we identified 356 pregnancies from 117 females, 52 of which resulted in fetal loss.

**Fig 1:**
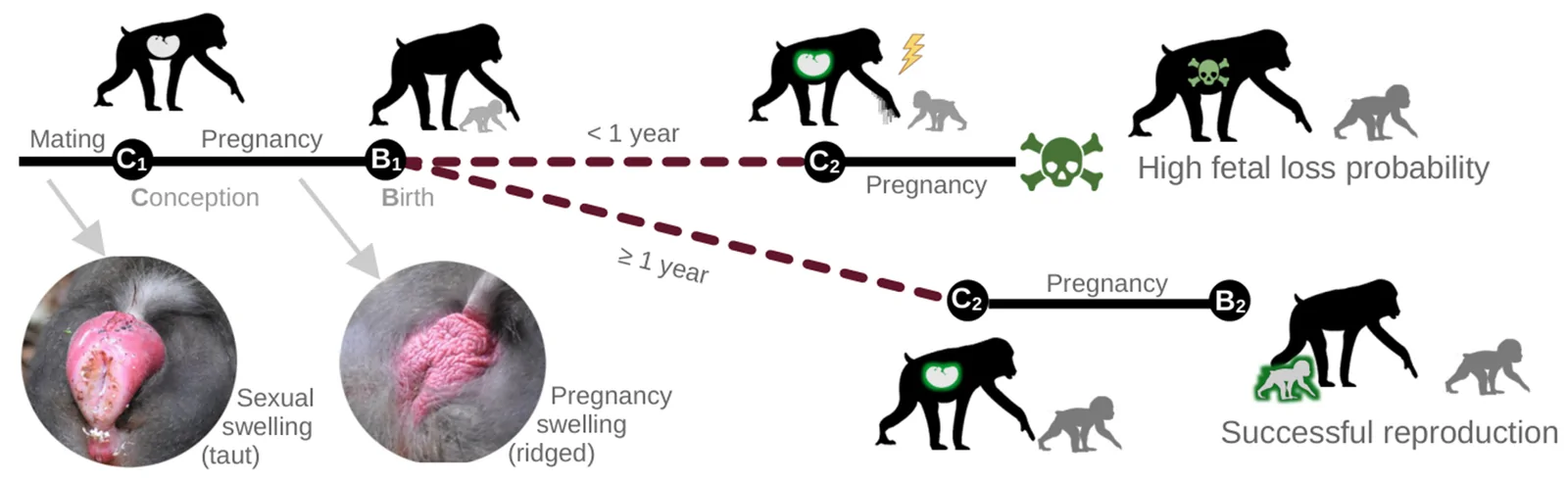
Mandrill timeline of reproduction with hypothesized pregnancy outcomes as a function of infant presence. Females who conceive while still caring for a dependent infant (top line) may exhibit a higher probability of fetal loss than those who conceive later, while not caring for a dependent infant anymore (bottom line). Pictures show perineal sexual and pregnancy swellings, which enable reliable identification of pregnancies.

## Results and discussion

### Fetal loss and infant presence

We first tested whether the presence of an infant (hereafter, ‘infant’ refers to a dependent offspring aged 0–12 months; Fig A in S1 Appendix) at the time of conception increases the probability of fetal loss (response variable 0/1) using a Bayesian Generalized Linear Mixed Model (GLMM) with a binomial distribution and a logit link function, controlling for different factors known to influence female reproductive patterns (see Text A in S1 Appendix for details and supplementary analyses). Pregnant females with an infant show a greater probability of fetal loss than those without, with a 100% posterior probability of a positive effect (i.e., all posterior draws for this variable exceed zero; estimate = 1.76; Fig 2A; Table A in S1 Appendix; 38 fetal losses, 278 pregnancies, 94 females). To test for sex-differential maternal investment, we restricted the dataset to females with an infant and fitted infant sex in the model, instead of infant presence. Fetal loss probability shows little association with infant sex, meaning that the probability of fetal loss is similar for mothers caring for sons or daughters (62% negative posterior probability; Table B in S1 Appendix; 26 fetal losses, 101 pregnancies, 41 females).

**Fig 2:**
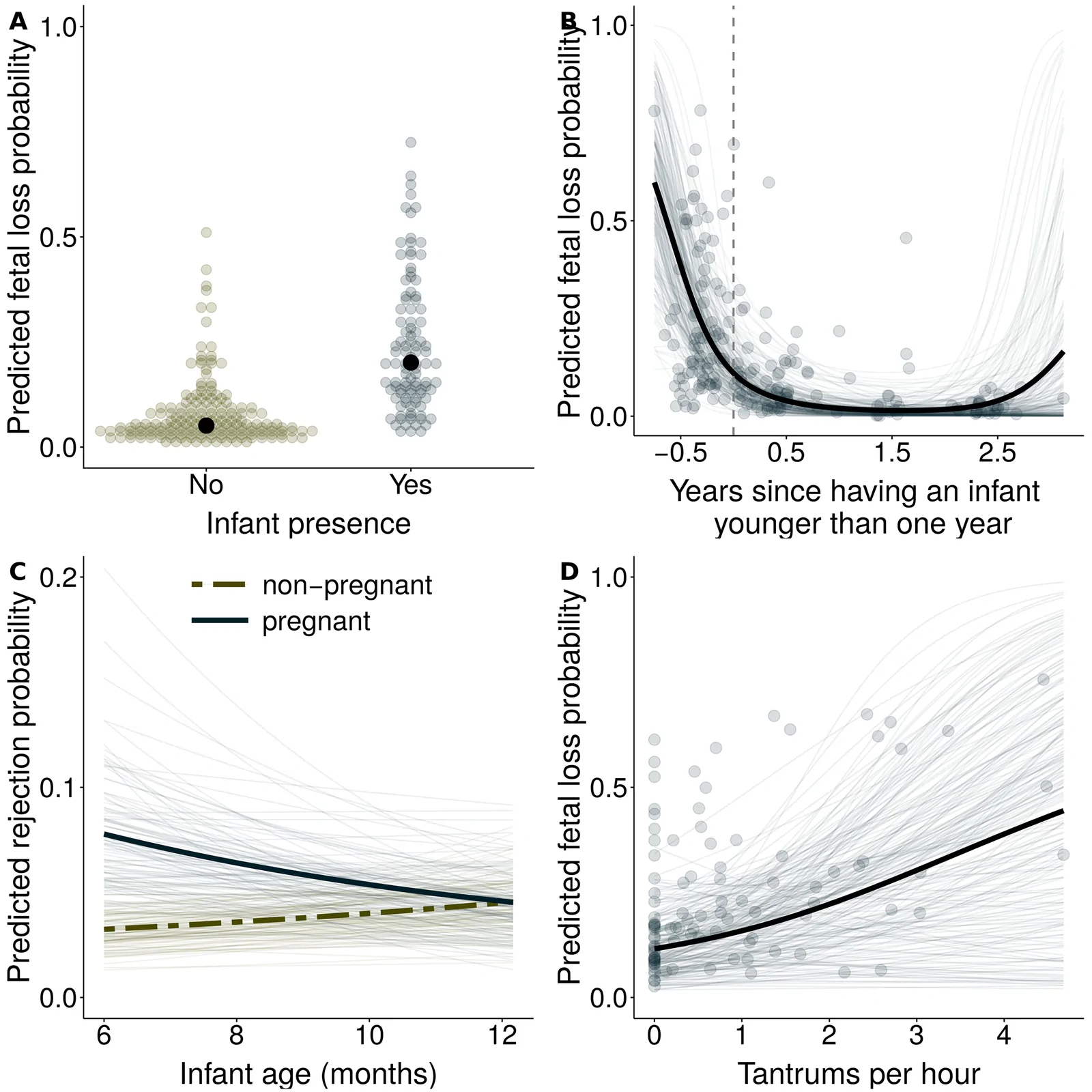
Fetal loss, infant presence, and mother-offspring behavioral conflict. Fetal loss probability as a function of **(A)** infant presence at the time of conception (black dots represent means; Table A in S1 Appendix), and **(B)** the time since the mother cared for an infant before conceiving again (vertical line at zero represents a case where a mother conceives when her previous infant, if still alive, reaches one year of age; Table D in S1 Appendix). **(C)** Maternal rejection in pregnant and nonpregnant females (Table H in S1 Appendix). **(D)** Fetal loss probability as a function of infant tantrum rate (Table I in S1 Appendix). Y-axes show predicted values of the GLMMs and faint lines represent uncertainty of predictions (200 posterior draws). The data underlying this figure can be found at: https://doi.org/10.5281/zenodo.21507883.

The impact of infant presence on fetal loss may indicate a depletion of maternal energetic reserves due to lactation and, more generally, maternal care, which are critical for another successful reproduction [14,15,21,22]. Alternatively, it may indicate that the time interval between two pregnancies is too short to allow a full recovery from the physiological and morphological changes or the energetic costs of the previous pregnancy [15]. To disentangle the costs of maternal care from those of pregnancy, we re-ran our model including, as an additional explanatory variable, the time between the previous and current conceptions (‘inter-conception interval’; C1–C2, Fig 1) representing the ‘recovery time’ since the previous pregnancy. While the effect of infant presence on fetal loss probability remains similar (100% positive posterior probability), posterior estimates suggest little relationship between inter-conception interval and fetal loss probability (linear: 54%, quadratic: 30%, positive posterior probability; Table C in S1 Appendix; 28 fetal losses, 166 pregnancies, 52 females). We further confirmed this result by replacing in our model the inter-conception interval with the time elapsed between the end of the previous pregnancy and the new conception (i.e., the time since the female last sustained a pregnancy; linear: 52%, quadratic: 26%, positive posterior probability). Therefore, infant presence appears to influence fetal loss independently of pregnancy costs, likely reflecting the costs of lactation or maternal care which are, indeed, usually greater than the costs of pregnancy, at least in primates [16].

We further confirmed the influence of infants on maternal pregnancy outcomes by running our analysis after replacing infant presence with a continuous variable representing the time elapsed between the presence of an infant and a new conception (i.e., the time since the female cared for an infant; range: -0.75–3.1 years; this variable allows to include females whose last offspring died before conception). Negative values corresponded to females who conceived while still caring for an infant (lower values correspond to younger infants). Positive values corresponded to females who conceived after the first birthday of their last offspring, representing the time that passed since they had to sustain potentially costly maternal care. Fetal loss probability is highest among females who conceive while still caring for young infants (x < 0 in Fig 2B), converges near zero for females who conceive a year after caring for their last infants (x≈1 in Fig 2B), and finally increases again for females who conceive more than two years after caring for their last infants (x > 2 in Fig 2B; U-shaped relationship; quadratic term estimate = 10.8, 96% positive posterior probability; Table D in S1 Appendix; 30 fetal losses, 202 pregnancies, 62 females). This late increase may reflect a common driver of reproductive pace and fetal loss. For example, poor health may both decelerate reproductive pace and increase fetal loss probability, as observed in humans and other mammals [23–26].

### Maternal care and mother-offspring conflict in pregnant and nonpregnant females

Given the impact of infant presence on fetal loss, we then tested whether maternal care decreases and/or mother-infant conflict intensifies when mothers are pregnant, potentially reflecting the energetic constraints associated with overlapping pregnancy and maternal care, changes in infant behavior, or both. Specifically, during an infant’s focal observation, we tested whether the probabilities of nursing and carrying are lower (maternal care), while the probabilities of infant tantrum (i.e., outbursts of distress characterized, in mandrills, by a set of distinctive vocalizations such as moaning and screaming), and maternal rejection (mother-to-infant refusal or, more rarely, aggression following an infant request) are greater when mothers are pregnant than when they are not (mother-offspring conflict; four GLMMs, each with a binomial distribution and a logit link function). Nursing, carrying and tantrum probabilities decline with infant age in a similar manner in pregnant and nonpregnant females (interaction between infant age and pregnancy status, nursing model: estimate = −0.002, 79% negative posterior probability; carrying model: estimate = 0.002, 82% positive posterior probability; tantrum model: estimate = 0.002, 78% negative posterior probability; Tables E–G in S1 Appendix; 8,819 focal observations of 314 infants). These weak associations suggest that infants likely have access to sufficient maternal care even when their mothers are pregnant. However, pregnant females reject their young infants more than nonpregnant females, suggesting intensified mother-infant conflict during pregnancy. Notably, the difference in rejection probabilities in pregnant and nonpregnant females decreases as infants approach one year of age (estimate = −0.005, 97% negative posterior probability; Fig 2C; Table H in S1 Appendix; 3,949 focal observations of 279 infants; see Text A in S1 Appendix for supplementary results).

### Maternal care, mother-offspring conflict and fetal loss probability

We then studied whether greater maternal care and/or intensified mother-infant behavioral conflict are associated with a greater probability of fetal loss (response variable 0/1), using a GLMM with a binomial distribution and a logit link function, and controlling for infant age and other relevant factors (care and conflict rates were calculated during the infant’s first year of life, see Materials and methods; Table I and Text A in S1 Appendix). Maternal nursing, carrying and rejection rates show little association with the probability of fetal loss (27%–49% positive posterior probability; Table I in S1 Appendix; 25 fetal losses, 98 pregnancies, 43 females). In contrast, greater infant tantrum rates are associated with an increased probability of fetal loss (estimate = 0.68; 96% positive posterior probability; Fig 2D). This result may reflect two nonexclusive mechanisms: either variation in maternal condition contributes to both greater tantrum rates and increased fetal loss probability, although in this case we would also expect changes in other proxies of maternal care; or infant behavior itself contributes to variation in fetal loss probability. The latter mechanism could represent an infant strategy delaying the birth of a younger sibling to prolong the period of exclusive maternal investment. Similar strategies have been described in other species, where offspring interfere with maternal sexual activity [27,28] or chemically suppress parental reproduction [29]. Disentangling these mechanisms would require detailed data on maternal condition, reproductive physiology, and behavioral interactions.

### Fetal loss and reproductive success

In several primate species, short inter-birth intervals are associated with increased infant mortality [10,19,30]. Similarly, in mandrills, we find a positive correlation between infant survival to 6 months and the interval since the previous birth (mean correlation coefficient = 0.17, 99% positive posterior probability; 45/382=12% of infants in the study population die before reaching six months of age). Under these conditions, fetal loss may limit the costs associated with short inter-birth intervals by preventing concurrent investment in two young offspring. Recent work suggests that female mammal physiology can indeed, to some extent, influence pregnancy onset or termination in ways that mitigate fitness costs (humans: [31]). For example, fetal loss may minimize the costs of producing infants who are likely to die due to female-female reproductive competition (banded mongooses, *Mungos mungo*; [32]) or male infanticide (geladas, *Theropithecus gelada*; [33]). Our results remind the latter case, as the probability of fetal loss for female mandrills is greater when the number of alpha male takeovers during the first month of pregnancy is greater (Fig 3; Table A in S1 Appendix: estimate = 0.41, 98% positive posterior probability; see also Tables B–D and I in S1 Appendix). Therefore, female mandrills, similarly to female geladas, may exhibit a physiological response to social conditions associated with elevated infanticide risk following male takeovers, inducing fetal loss to minimize investment into doomed reproductive efforts (‘spontaneous fetal loss’; [33,34]). Alternatively, this result may reflect increased female stress or feticide risk after male takeovers [35–37].

**Fig 3:**
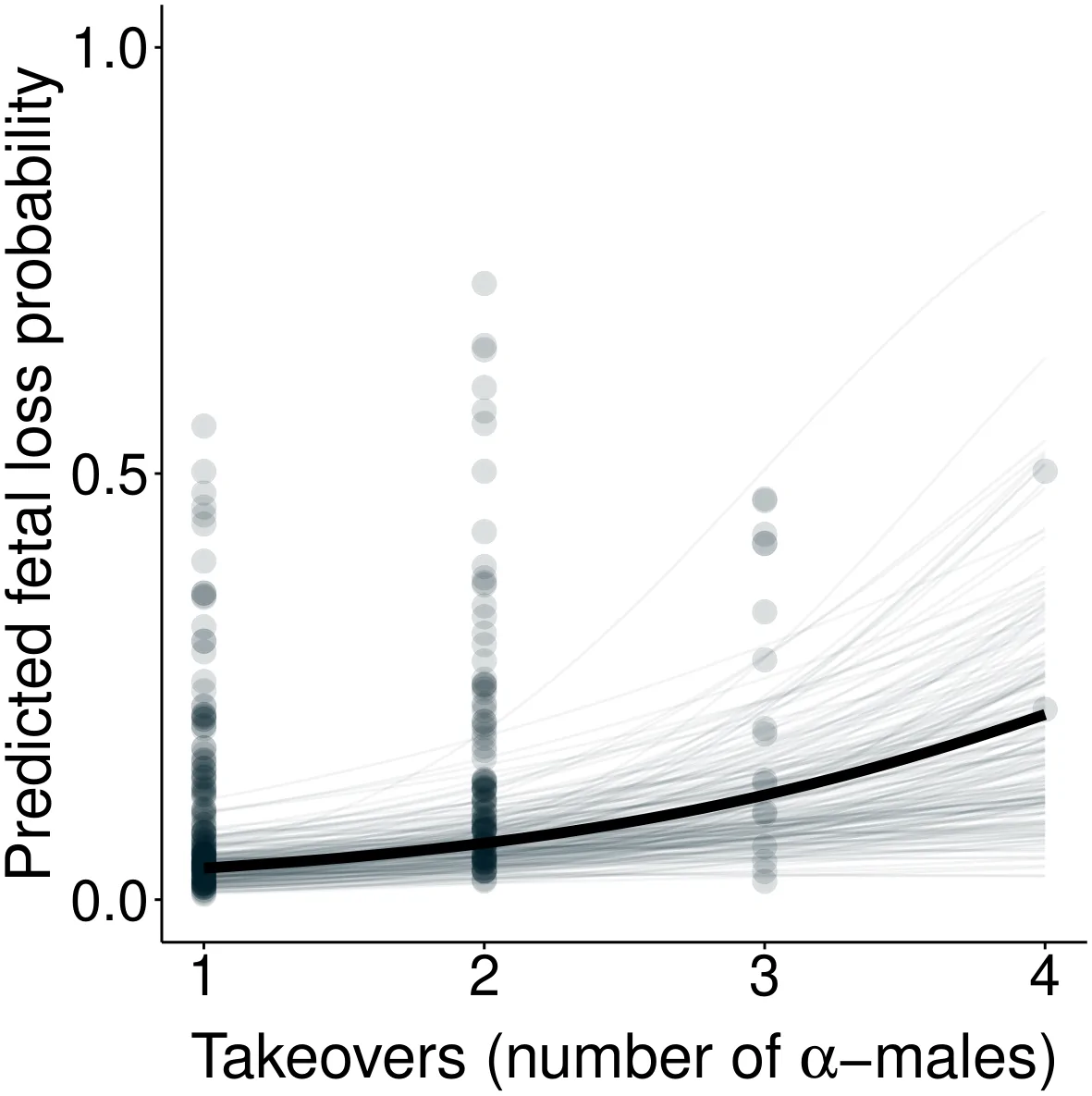
Fetal loss and male takeovers. Fetal loss probability as a function of the number of males that claimed the alpha rank in the male hierarchy during the first month of pregnancy (Table A in S1 Appendix). Y-axis shows predicted values of the GLMM and faint lines represent uncertainty of prediction (200 posterior draws). The data underlying this figure can be found at: https://doi.org/10.5281/zenodo.21507883.

We examined whether fetal loss could be an adaptive mechanism improving female reproductive success, and whether this mechanism may function not only as a response to increased infanticide risk but also in response to maternal energetic depletion or mother-offspring conflict which may have fatal consequences for offspring. Specifically, we compared the medium-term reproductive success of female mandrills who experienced fetal loss with those whose infants died soon after birth (before 6 months of age; for a similar approach, see [33]). We quantified medium-term reproductive success, as the number of offspring who reached 6 months of age during a period equal to two median inter-birth intervals (3.1 years) starting from a successful reproductive event (defined as the onset of a pregnancy resulting in an infant surviving to 6 months; see Fig 4). We chose a 6-month survival threshold because female mandrills can resume cycling roughly 6 months after birth (see also [20]). We used a window of two median inter-birth intervals to standardize comparisons: females with consecutive successful reproductive events are expected to produce at least two surviving offspring, whereas those experiencing fetal loss or early infant death are expected to produce only one or, at most, two offspring. Females who experience fetal loss (Fig 4, bottom row) conceive sooner, often within the same mating season, than females whose infants die before 6 months of age (Fig 4, middle row), and consequently produce more offspring on average (1.8 ± 0.4 versus 1.4 ± 0.5; 100% posterior probability; Fig 4). Moreover, females who experience fetal loss produce a statistically similar number of surviving infants with females who reproduce uninterrupted (Fig 4, top row; 2.1 ± 0.4; 50% posterior probability). Finally, we confirmed these results by using inter-birth interval length between two infants who survive to 6 months of age as an alternative measure of medium-term reproductive success. Females who experience fetal loss between two surviving infants exhibit a shorter average inter-birth interval (710 ± 119 days [±s.d.]; Fig B in S1 Appendix, bottom row) than females who experience infant death between the production of two surviving infants (872 ± 312; Fig B in S1 Appendix, middle row; 89% posterior probability), and a longer average inter-birth interval than females who produce two survival infants uninterrupted (IBI: 578 ± 148; Fig B in S1 Appendix, top row; 100% posterior probability).

**Fig 4:**
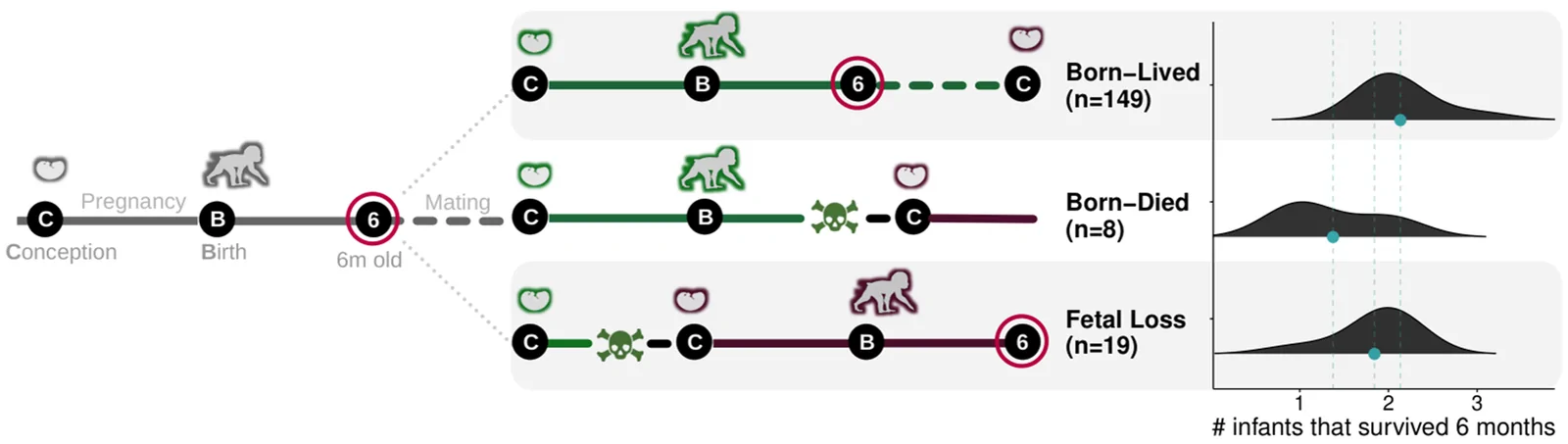
Number of infants who survived 6 months within a period of two median inter-birth intervals (3.1 years; ‘medium-term reproductive success’) as a function of the outcome of two consecutive pregnancies. We observed 149 cases where a pregnancy resulted in a birth of an infant who survived 6 months (top row, 'Born-Lived'), 8 cases where a pregnancy resulted in a birth of an infant who died before 6 months (middle row, 'Born-Died'), and 19 cases where a pregnancy resulted in a fetal loss (bottom row, 'Fetal Loss'). The minimum number of surviving infants is always one since we included only cases where the initial reproductive event was successful. Blue dots and vertical lines within density plots represent means. The data underlying this figure can be found at: https://doi.org/10.5281/zenodo.21507883.

Our results suggest that fetal loss, while costly in the short-term (i.e., a single reproductive event), may mitigate medium-term fitness consequences (e.g., over a period including two consecutive reproductive events) arising when females are physiologically unable to sustain short inter-birth intervals. Thus, fetal loss could be a key determinant of reproductive success, partly contributing to the large variation in lifetime reproductive success observed among females in this population (12 females who were alive and of reproductive age throughout the 11-year study period produced between 2 and 9 living offspring). More broadly, given the substantial energetic costs of lactation and maternal care in mammals, and particularly in primates [12,13,16], fetal loss may reflect a physiological process that limits reproductive costs under energetically constrained conditions.

### Conclusions

Fetal loss is widespread, observed in 10–20% of pregnancies in human and nonhuman primates (14.6% in our study population; [38–40]) and entails substantial costs, such as squandered time and energy, as well as potential health consequences for females, including infection or psychological complications that may jeopardize future pregnancies or even survival [25]. Thus, understanding the evolutionary and physiological determinants of fetal loss may have implications extending beyond life-history theory, informing both animal ecology and human reproductive health [41]. Our findings suggest that fetal loss may represent an adaptive consequence of female attempts to accelerate reproductive pace by sustaining concurrent pregnancy and maternal care. Maternal energetic exhaustion—potentially amplified by mother-offspring conflict—may occasionally increase fetal loss probability, while fetal loss itself may partly buffer the fitness costs of rapid reproduction. Our findings further highlight the need to investigate the physiological pathways linking fetal loss to maternal condition, particularly the mechanisms by which trade-offs between offspring quality and quantity influence reproductive pace. They finally suggest that the lower fecundity observed in females with young offspring across many mammals may not be explained solely by lactational amenorrhea but could also stem from a higher probability of fetal loss [42,43]. Altogether, our study refines our understanding of the physiological and life-history constraints that shape reproductive pace.

## Materials and methods

### Study system

Mandrills are seasonal breeding primates of central Africa who live in large polygynandrous groups of philopatric females and their young [44]. Male mandrills generally immigrate in a social group early in the mating season and often emigrate the following birth season [45]. We studied a large population of mandrills habituated to human presence who live in the surroundings of the Lékédi Park in Southern Gabon and is monitored daily by the Mandrillus Project since 2012. The group was founded by captive female mandrills released from the Centre Interdisciplinaire de Recherches Médicales de Franceville (CIRMF, Gabon), in 2002 and 2006 [46]. We used 11 years (from 2012 to 2023, except 2020 due to the COVID19 pandemic) of detailed life-history, behavioral, and reproductive data collected by trained observers blind to this study’s questions. At the end of the study, the group consisted of c.a. 300 individuals, including only four females born in captivity.

### Reproductive seasonality and female swellings as indicators of reproductive state

Alternating mating and birth periods last roughly 6 months in mandrills [20]. Females usually conceive every other mating season and occasionally in consecutive mating seasons while still caring for dependent infants (median inter-birth interval: 566 days, range 323–1,024; [20]). Based on patterns of reproduction and births in our study population, we considered as mating season the period spanning from April 1st to September 30th, and as birth season the following 6 months (October to March; [20]). Female mandrills generally exhibit estrus cycles during the mating seasons. During each cycle, their perineal skin inflates gradually, reaches maximal size around ovulation and finally deflates in a few days [47]. Observers assigned daily a score from 0 to 3 by increments of 0.5 to quantify the size of the sexual swelling of each female. In addition, they recorded the presence of a distinctive, small, ridged swelling that female mandrills exhibit a few weeks after conception (pregnancy swellings: Fig 1). We used combined information of sexual and pregnancy swellings to identify fetal loss; that is, confirmed pregnancies that did not result in a birth of a living infant. Following a fetal loss, females generally resume cycling within the two next weeks (unpub. data). Given that pregnancy swellings appear a few weeks after conception, our data may not include fetal losses occurring at the earliest stages of pregnancy. The earliest fetal loss we observed occurred 1.3 months after conception, and fetal loss was observed on average 4.1 ± 1.4 months after conception. Notably, including earlier fetal losses is unlikely to alter our conclusions, as these cases would involve younger dependent infants, a context in which maternal exhaustion or mother-infant conflict may be even more pronounced than in females experiencing fetal loss later.

### Predictors of fetal loss probability in female mandrills

**Infant presence and age.** We defined infants as individuals under one year of age. We knew the exact age of 156 infants, and estimated age with a maximum error of 10 days for 125, 30 days for 20, 60 days for one, and 90 days for one infant. When we run our analyses including only infants with an age error of 10 days or less, our results remain qualitatively similar.

**Maternal care and mother-offspring behavioral conflict.** We used all 8,819 focal observations (531 hours of observation) performed on the 314 study infants (0–12 months old) born to the 117 study females to quantify two behavioral proxies of maternal care (nursing and carrying) and two behavioral proxies of mother-offspring conflict (infant tantrum and maternal rejection [pushing, slapping, grasping/hitting, and biting]) [48,49]. We used a presence/absence approach, that is, we identified the infant focal observations which included at least one record of nursing/carrying/tantrum/rejection, rather than the number or duration of events, because these behaviors occur in short variable bouts that are difficult to count or measure precisely, and they are often non independent. For example, infants may momentarily pause during nursing or tantrum, mothers may momentarily stop carrying their infants, and they may use a combination of rejecting behavior sequentially.

**Maternal age.** Female age may influence fetal loss probability because of reproductive immaturity (too young ages) and/or senescence (too old ages; humans: [25]). We knew the exact age of 41 study female mandrills and we estimated the age of the remaining females based on body condition and patterns of tooth eruption and wear [50]. We considered as adult those females aged four years and above.

**Maternal rank.** Female dominance rank may predict fetal loss probability as it also predicts inter-birth interval length in female mandrills [20]. We inferred female ranks using approach-avoidance interactions obtained both from focal sampling (3,116 hours of adult female observations) and ad libitum behavioral observations. We used an Elo-rating algorithm (R package EloRating; [51]) which assigned to adult females present at the beginning of the study initial Elo scores of 1,000, to females entering the hierarchy the score of the lowest-ranking individual at the entrance day, and to all interactions equal intensity (k = 100). We standardized the resulting Elo-scores (ranks) so that, on a given day, the highest-ranking female had a score of one and the lowest-ranking female had a score of zero.

**Social bonds.** Female-female mandrill bonds may influence fetal loss probability because they are linked to both reproductive success [52] and dominance rank [53]. We used as a proxy of female social bonds, the number of adult female maternal kin, calculated as the sum of maternal half-sisters, daughters, and mother present in the group at the time of conception.

**Distance to conception peak.** Female mandrills who conceive further from a conception peak exhibit a greater fetal loss probability [20]. Based on reproductive patterns observed in the study population, we determined March 1st as the transition date between ‘conception cohorts’. We used conceptions for which the date was known with a maximum of 30 days of uncertainty to calculate conception peaks (as per [20]): we converted conception dates into radian angles and calculated a mean direction (μ) for each cohort using the function *circ.summary* of R package *CircStats* [54]; then, we transformed μ into a calendar date (‘conception peak’) and for each conception, we calculated its absolute distance from the conception peak (number of days, irrespective of whether it is before or after the peak) of the corresponding cohort.

### Statistical analysis

**Fetal loss and infant presence.** We ran a Bayesian GLMM with a binomial distribution and a logit link function, to test if the probability of fetal loss is influenced by the presence of an infant (offspring 0−12 months old) at the time of conception (0/1). In this model, we further tested for female rank, female age and its quadratic term (or parity: parous versus nulliparous; alternative model), the number of adult female maternal kin present in the group at the time of conception, female foraging rate, the number of alpha male takeovers during the first month of pregnancy, and the absolute distance (in days) of female’s conception from the conception peak that year. We fitted the identity of the mother and the year of conception as two random factors (intercepts). We also confirmed the results of this analysis by running our model including the time since the female had a dependend infant instead of infant presence. Female age was not strongly correlated neither with her rank (mean correlation coefficient = −0.06, 89% negative posterior probability), nor with the time since she had a dependent infant (mean correlation coefficient = 0.09, 91% positive posterior probability). However, female rank was correlated with infant presence (mean correlation coefficient = 0.26, 100% positive posterior probability) and the time since she had a dependent infant (mean correlation coefficient = −0.25, 100% negative posterior probability), consistent with observations that high-ranking females have shorter inter-birth intervals than low-ranking females. Yet, when we exclude from our analysis the highest-ranking females (with standardized Elo-rating>0.8), our results remain similar, that is, pregnant females with a dependent infant show a greater probability of fetal loss than those without, with a 99% posterior probability of a positive effect.

**Fetal loss and recovery time since the previous pregnancy.** We tested if fetal loss probability increases due to physiological constraints related to limited recovery time rather than the presence of an infant, by running a modified version of the model on infant presence adding the inter-conception interval (number of days since previous conception; C1–C2 on Fig 1) and its quadratic term as explanatory variables. Because a female cannot have a dependent infant (0–12 months) at the time of a new conception if this conception occurs much later than her previous conception, we restricted this model to cases where the inter-conception interval was ≤556 days (1 year plus the maximum pregnancy length in our study population; 365 + 190 days; [20]). This model included females who experienced a previous pregnancy but do not necessarily have an infant present at the time of the second conception due to infant death or fetal loss. Finally, we confirmed the results of this analysis by running our model including the time elapsed between the end of the previous pregnancy and the new conception instead of the inter-conception interval.

**Maternal care and mother-offspring conflict in pregnant and nonpregnant females.** We analyzed 3,949 focal observations of 279 infants to test if maternal care to the infant decreases and mother-infant conflict intensifies during pregnancy. In this analysis, we used only data on infants older than 6 months (6–12 months old offspring), as data on pregnant mothers with very young infants are scarce (the minimum age at which an infant had a pregnant mother was ≈3 months). Specifically, we ran four GLMMs with binomial distributions and logit link functions to test if nursing (0/1; response variable: did the mother nurse her infant?), carrying (0/1; response variable: did the mother carry her infant?), infant tantrum towards the mother (1/0; response variable: did the infant throw a tantrum towards its mother?), and maternal rejection (0/1; response variable: did the mother reject her infant?) are more likely to occur during a given focal observation of the infant when mothers are pregnant than when they are not pregnant. We fitted an interaction between pregnancy status and infant age to test if the effect of infant age on mother-infant interactions depends on mother’s pregnancy status. In these models, we further controlled for infant sex, mother age, and rank. We fitted the identity of the infant, the identity of the mother and the year of observation as three random factors (intercepts).

**Maternal care, mother-offspring conflict and fetal loss probability.** We analyzed 8,819 focal observations of 314 infants (0–12 months old offspring) to test if the probability of fetal loss is greater when maternal care to the infant (nursing and carrying) and mother-infant conflict (tantrum and maternal rejection) during the first year of infant’s age are greater. We ran one GLMM with a binomial distribution and a logit link function to test the influence of all four behavioral proxies on fetal loss probability (0/1). We calculated behavioral rates as the number of focal observations in which nursing/carrying/tantrum/rejection was recorded at least once, divided by the total number of hours the infant was observed. We divided by the hours of observation rather than the number of focal observations because focal observations are often interrupted when observers lose their focal animals in the dense rainforest. In this model, we further controlled for the time since the birth of the infants (number of days between the infant’s birth and the new conception), mother rank, age and its quadratic term, the number of adult female maternal kin in the group at the time of conception, female foraging rate, the number of alpha male takeovers during the first month of pregnancy, and the absolute distance (in days) of female’s conception from the conception peak of that year. We fitted the identity of the mother and the year of conception as two random factors (intercepts).

**Fetal loss and reproductive success.** We tested if females who experience fetal loss show greater medium-term reproductive success than females who give birth to infants who die young. Specifically, for any given pregnancy (‘first pregnancy’; gray in Fig 4) that resulted in the birth of an infant who survived to 6 months of age, we calculated the reproductive success of the female as the number of her infants who survived to 6 months of age, within a period of two median inter-birth intervals (3.1 years) starting from the conception day of the first pregnancy. Given that we included only cases where the first pregnancy resulted in a successful reproductive event (i.e., infant surviving to 6 months), the minimum medium-term reproductive success was equal to one infant. We used a Bayesian alternative to the traditional *t* test to compare the mean and distribution of the number of surviving infants of females who experienced fetal loss during their second pregnancy (green in Fig 4) and the number of surviving infants of females whose second pregnancy resulted in an infant who died before 6 months of age.

We also tested if females who experience fetal loss show shorter inter-birth intervals between two surviving infants than females who give birth to infants who die young. Specifically, for any given pregnancy (‘first pregnancy’) resulting in the birth of an infant who survived to 6 months of age, we calculated the inter-birth interval to the next birth also resulting in the birth of an infant who survived to 6 months. We used a Bayesian alternative to the traditional *t* test to compare the mean and distribution of inter-birth intervals of females who experience a fetal loss between two births of surviving infants, those who experienced an infant death between two births of surviving infants, and those who experienced no interruption. We excluded from this analysis the following cases: (i) two cases where a female lost both a fetus and an infant before producing an infant who survived to 6 months (in one of these two cases, the female lost two fetuses and one infant), and (ii) five cases where a female lost two fetuses before producing an infant who survived to 6 months. We did not observe cases where a female lost two infants before producing an infant who survived to 6 months.

We ran all statistical tests using a Bayesian framework in R version 4.3.3 [55]. We used function *brm* of package *brms* (version 2.22.0) to run the GLMMs [56]. We used function *scale* from base R to standardize continuous variables fit as predictors in the GLMMs. We used informative priors (normal (0,1)) for continuous variables and four chains with 2000 iterations in each. The first 1,000 iterations were discarded as warmup, retaining 4,000 post-warmup draws with no thinning. All Rhat values were <1.01, indicating model convergence. We assessed model fit using posterior predictive checks with functions/plots *pairs* and *pp_check* from package *brms*. We reported posterior means of population-level (fixed) effects, which represent estimates averaged over the random effect structure (marginal effects). We used function *correlationBF* of package *BayesFactor* (version 0.9.12-4.7) to calculate correlation coefficients and assess multicollinearities [57]. We used function *BESTmcmc* of package *BEST* (version 0.5.4) to compare group means and distributions. We consider an effect to provide little evidence of association when fewer than 89% of posterior samples share the same sign, that is, when less than 89% are positive and less than 89% are negative. Finally, we used package ggplot2 (version 4.0.2) [58] to visualize our results.

This study was based only on noninvasive observational protocols, it was approved by an authorization from the CENAREST institute (permit number, AR15/24/MESRSIT/CENAREST/CG/CST/CSAR), and followed all applicable guidelines for the care and use of animals.

## Supporting information

S1 Appendix Supplementary Appendix (Text A, Figures A & B, and Tables A–J). **Text A in S1 Appendix.** Supplementary Text. **Fig A in S1 Appendix.** Infant nursing probability as a function of infant age. **Fig B in S1 Appendix.** Inter-birth intervals between the births of two infants that survived to 6 months, without interruption, with an infant death or with a fetal loss in between. **Table A in S1 Appendix.** Results from the GLMM testing for the influence of infant presence on fetal loss probability. **Table B in S1 Appendix.** Results from the GLMM testing for the influence of infant presence on fetal loss probability. **Table C in S1 Appendix.** Results from the GLMM testing for the influence of infant presence and inter-conception interval on fetal loss probability. **Table D in S1 Appendix. Table E in S1 Appendix.** Results from the GLMM testing for the influence of infant age and mother’s pregnancy status on the probability of nursing. **Table F in S1 Appendix.** Results from the GLMM testing for the influence of infant age and mother’s pregnancy status on the probability of carrying. **Table G in S1 Appendix.** Results from the GLMM testing for the influence of infant age and mother’s pregnancy status on the probability of tantrum. **Table H in S1 Appendix.** Results from the GLMM testing for the influence of infant age and mother’s pregnancy status on the probability of rejection. **Table I in S1 Appendix.** Results from the GLMM testing for the influence of mother-infant interaction rates on fetal loss probability. **Table J in S1 Appendix.** Results from the GLMM testing for the influence of male sexual coercion (intimidation) to the female on fetal loss probability.
(PDF)
