# Supplementary material for: Infant presence and mother-infant conflict predict fetal loss in wild mandrills
Source: PLoS Biol. 2026 Nov 3;24(11):e3003970. doi: 10.1371/journal.pbio.3003970 (PMC13730340; doi:10.1371/journal.pbio.3003970)
Supplement: Supplementary file 1 [file pbio.3003970.s001.pdf]

Supporting information for the article:
Infant presence and mother-infant conflict predict fetal loss in wild mandrills

Nikolaos Smit[1,2,*], Berta Roura-Torres[3,4,5], Axelle Delaunay[2], Alice Baniel[3], Elise Huchard[3], Marie J. E. Charpentier[3]

1 Max Planck Institute for Evolutionary Anthropology, Deutscher Platz 6, 04103 Leipzig, Germany
2 Department of Biology, University of Turku, Vesilinnantie 5, 20014 Turku, Finland
3 Institute of Evolutionary Sciences of Montpellier (ISEM), University of Montpellier, CNRS, IRD, EPHE, Montpellier, France
4 Behavioral Ecology and Sociobiology Unit, German Primate Center, Leibniz Institute for Primate Research, Kellnerweg 4, 37007 Göttingen, Germany
5 Department of Sociobiology/Anthropology, Johann-Friedrich-Blumenbach, Institute of Zoology and Anthropology, Georg-August University Göttingen, Kellnerweg 6, 37077 Göttingen, Germany

* nismit@utu.fi

Text A

In our analyses, we controlled for several factors known or hypothesized to influence reproductive success (and more specifically, fetal loss) and mother-infant interactions. Results from these control factors are described in the text and shown in the tables below, following each result section of the main text. Sample sizes (see Tables' legends) differ among models due to variation in data availability across variables. We also repeat here the model description to facilitate comprehension of the full models and their results.

Supplementary results from the analysis in the main text

Fetal loss and infant presence

Model description (similar to the main text). We ran a Bayesian Generalized Linear Mixed Model (GLMMs) with a binomial distribution and a logit link function, to test if the probability of fetal loss is influenced by the presence of an infant (offspring 0-12 months old) at the time of conception (0/1). In this model, we further tested for female rank, female age and its quadratic term (or parity: parous vs. nulliparous; alternative model), the number of adult female maternal kin present in the group at the time of conception, female foraging rate, the number of alpha male takeovers during the first month of pregnancy, and the absolute distance (in days) of female's conception from the conception peak that year. We fitted the identity of the mother and the year of conception as two random factors (intercepts; Table A in S1 Appendix). Then, we run our analysis replacing infant presence with infant sex (Table B in S1 Appendix). We also tested if fetal loss probability increases due to physiological constraints related to limited recovery time rather than the presence of an infant, by running a modified version of our model on infant presence including the inter-conception interval (number of days since previous conception; C1-C2 on Fig 1) and its quadratic term as

explanatory variables (Table C in S1 Appendix). Finally, we ran an alternative model replacing infant presence with the time since the female had a dependent infant (Table D in S1 Appendix).

Results. A lower female rank and a longer time distance from the conception peak of the respective mating season are both associated with a greater fetal loss probability (Table A-D in S1 Appendix), confirming previous results in our study population [1]. The latter result is likely driven by the females who conceive late within a mating season (later than the conception peak) and give birth late in the following birth season, meaning that their infants will be very young and still vulnerable to infanticide at the onset of the next mating season, when males immigrate into the group, inducing male hierarchy instability and increasing infanticide risk [1]. This interpretation is also in line with our result on alpha male takeovers (see main text). The number of maternal kin, female age, and foraging rate have a weak effect on fetal loss probability (Table A-D). When we run our model testing for infant presence including female parity instead of age, nulliparous females (vs. parous females; estimate= -1.09; 97% posterior probability) show greater fetal loss probability, potentially suggesting immature reproductive physiology. Finally, when excluding the 16 captive-born females from our dataset, the effect of infant presence on fetal loss probability remains similar (99% positive posterior probability), suggesting that our results are not driven by the early life experiences of these females.

Maternal care and mother-offspring conflict in pregnant and non-pregnant females

Model description (similar to the main text). We ran four GLMMs with a binomial distribution and a logit link function to test if nursing (0/1; response variable: did the mother nurse her infant during a given focal observation of the infant?), carrying (0/1; response variable: did the mother carry her infant during a given focal observation of the infant?), infant tantrum towards the mother (1/0; response variable: did the infant throw a tantrum towards its mother during a given focal observation?), and maternal rejection (0/1; response variable: did the mother reject her infant during a given focal observation of the infant?) are more common when mothers are pregnant than when they are non-pregnant. We fitted an interaction between pregnancy and infant age to test if infant age influences mother–infant interactions differently depending on the mother's pregnancy status. In these models, we further controlled for infant sex, mother age and rank. We fitted the identity of the infant, the identity of the mother and the year of observation as three random factors (intercepts).

Results. In the model testing for infant nursing, older females appear to nurse their infants less than younger ones (Table E). In the model testing for infant tantrum, infants of younger females throw tantrums more often than infants of older females (Table G). In the model testing for mother rejection, younger females appear to reject their infants more, and females reject more their daughters than their sons (Table H). All other effects have a little influence on mother-infant interactions in the four models on nursing, carrying, rejection, and tantrum probabilities (see Tables S5-S8 in S1 Appendix for details).

Maternal care, mother-offspring conflict and fetal loss probability

Model description (similar to the main text). We ran a GLMM with a binomial distribution and a logit link function to test if the probability of fetal loss is influenced by mother-infant interactions during the infant's first year of life. Specifically, we fitted as explanatory variables the rates of nursing, carrying, tantrum and rejection. In this model, we further controlled for infant age (number of days between the infant's birth

and the new conception), mother rank, age and its quadratic term, the number of adult female maternal kin in the group at the time of conception, female foraging rate, the number of alpha male takeovers during the first month of pregnancy, and the absolute distance (in days) of female's conception from the conception peak of that year. We fitted the identity of the mother and the year of conception as two random factors (intercepts).

Results. Results of this model further confirm that females of lower rank experience greater fetal loss probability, while the number of maternal kin, female age, and female foraging rate have a little influence on fetal loss probability (Table I). A shorter period between infant birth and a new conception is also linked to a greater fetal loss probability (Table I). Notably, in this restricted model, the days to conception peak have a weaker influence on fetal loss probability (Table I).

Supplementary analysis on sexual coercion

Sexual coercion (intimidation). Male mandrills use sexual coercion to increase their mating success with targeted females. Particularly, they intimidate females prior to estrus cycle, increasing mating rates during that cycle [2]. Such male behaviors might be associated with greater fetal loss probability either through increased injury risk (injured females may experience fetal loss as a byproduct) or through altered mating patterns (e.g., mating with low-quality males due to coercion that females would otherwise avoid [2]). We quantified sexual intimidation rate as per [2,3]: for each conception, we counted the total number of adult male aggressive events (males aged >9 years) towards the female, recorded during a period starting on the first immigrant male's arrival in the group that year ('onset of the mating season') and ending on the first day of the swelling phase of the female's estrus cycle. Then, we divided the number of these aggressive events by the total time of focal observations. For each mating season, we considered as immigrant males those males who were first censused in the group during that mating season (April-September) while they were not censused in the group during the three preceding months (January-March; [2,3]). While not all male-to-female aggression during the 'coercion window' necessarily reflects sexual coercion, we have previously shown that such aggression ('sexual intimidation'), and not aggression observed during the swelling phase, increases male perpetrator's mating success with the female target [2]. We tested whether male sexual intimidation influences the probability of fetal loss by running the initial model on infant presence, adding as an explanatory variable the coercion rate the mother received before conception. Sexual intimidation is weakly correlated with the number of alpha males in the group during the first month of pregnancy (mean correlation coefficient=-0.06, 15% positive posterior probability), meaning that we could add both in this model. Coercion rate has a little influence on fetal loss probability (estimate= -0.22; 66% negative posterior probability; Table J in S1 Appendix).

References

1. Dezeure J, Charpentier MJE, Huchard E. Fitness effects of seasonal birth timing in a long-lived social primate living in the equatorial forest. Animal Behaviour. 2022 Mar;185:113-26. doi:10.1016/j.anbehav.2022.01.002.

2. Smit N, Baniel A, Roura-Torres B, Amblard-Rambert P, Charpentier MJE, Huchard E. Sexual coercion in a natural mandrill population. Peer Community Journal. 2022 Jun;2:e36. doi:10.24072/pcjournal.134.

3. Smit N, Dezeure J, Sauvadet L, Huchard E, Charpentier MJE. Socially bonded females face more sexual coercion in a female-philopatric primate. iScience. 2023 Oct;26(10):107358. doi:10.1016/j.isci.2023.107358.

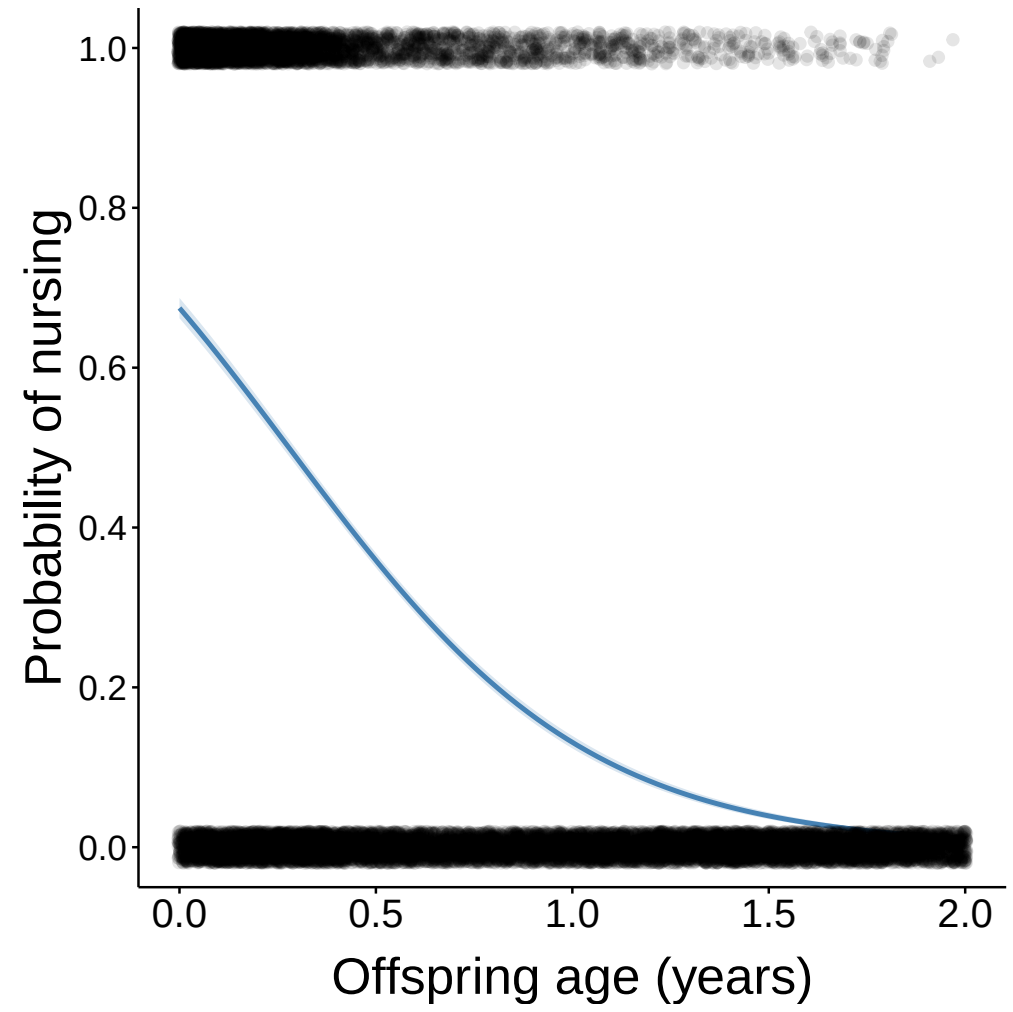

Fig A. Infant nursing probability as a function of infant age. Each datapoint corresponds to a focal observation of an individual between one and two year old. The decline around one year of age months reflects the weaning process. The data underlying this figure can be found at: `https://doi.org/10.5281/zenodo.21507883` (file: Data_Online_Nursing)

Table A. Results from the GLMM testing for the influence of infant presence on fetal loss probability. This model ran on 278 pregnancies of 94 females, including 38 that resulted in a fetal loss. Standard deviations (SD) of random effects indicate the degree of variation attributable to each grouping factor. $R^2 = 0.23 \pm 0.05$ (estimate $\pm$ error).

Response variable: Fetal loss probability

Parameter	Estimate	Est.Error	Positive draws (%)
Intercept	-3.033	0.466	0.0
Female kin	0.028	0.219	55.3
Alpha male takeovers	0.411	0.201	97.9
Mother's age	-0.390	0.995	34.5
Mother's age^2	0.300	0.994	62.2
Mother's rank	-0.625	0.231	0.4
|Days to conception peak|	0.394	0.175	98.5
Foraging rate	0.276	0.225	89.1
Infant presence	1.756	0.449	100.0
SD (Mother)	0.460	0.351	NA
SD (Year)	0.746	0.440	NA

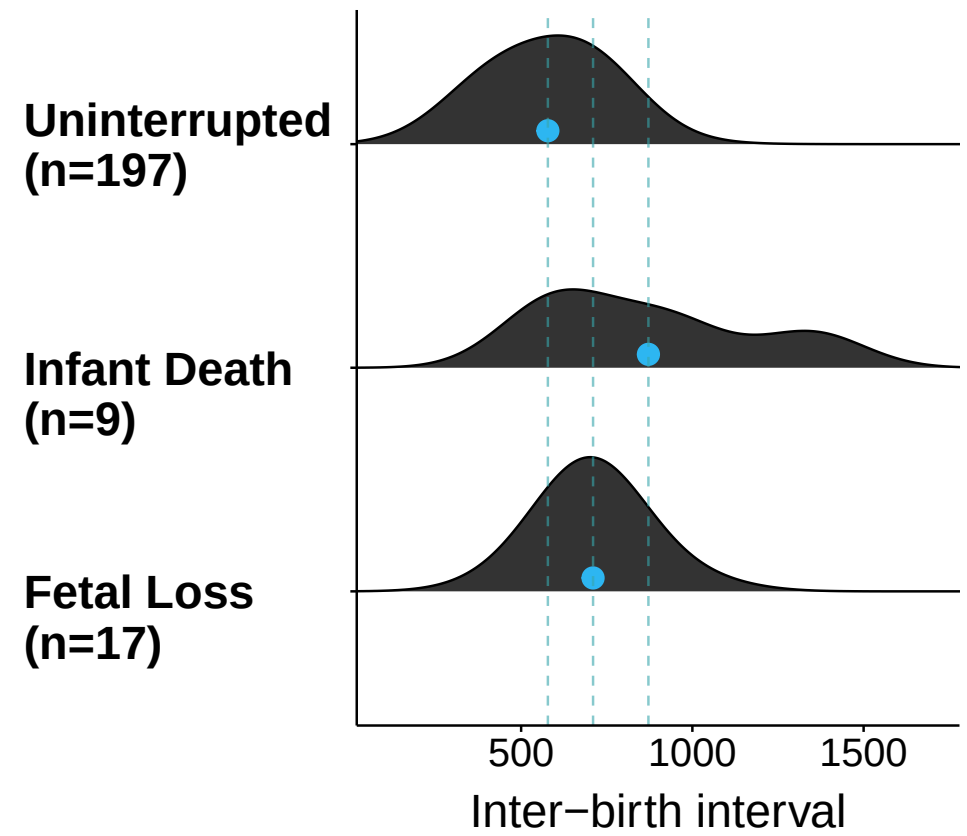

Fig B. Inter-birth intervals between the births of two infants that survived to six months, without interruption, with an infant death or with a fetal loss in between. In 197 cases no other pregnancy was observed between the two pregnancies that resulted in surviving infants (top row, uninterrupted; mean±s.d. of inter-birth interval [IBI]: 578±148). In 9 cases, we observed an infant death between the two successful pregnancies (middle row, Infant Death; IBI: 872±312). In 17 cases, we observed a fetal loss between the two successful pregnancies (bottom row, Fetal Loss; IBI: 710±119). Blue dots within density plots represent means. The data underlying this figure can be found at: `https://doi.org/10.5281/zenodo.21507883`. (file: Data_Online_Fitness)

Table B. Results from the GLMM testing for the influence of infant sex on fetal loss probability. This model ran on 101 pregnancies of 41 females, including 26 that resulted in a fetal loss. Standard deviations (SD) of random effects indicate the degree of variation attributable to each grouping factor. $R^2 = 0.37 \pm 0.09$ (estimate $\pm$ error).

Response variable: Fetal loss probability

Parameter	Estimate	Est.Error	Positive draws (%)
Intercept	-2.097	0.905	0.4
Female kin	0.266	0.351	78.7
Alpha male takeovers	0.747	0.406	97.4
Mother's age	-0.060	0.992	46.8
Mother's age^2	0.055	0.989	52.4
Mother's rank	-0.966	0.416	1.1
|Days to conception peak|	0.019	0.011	97.1
Foraging rate	-0.051	0.370	44.9
Infant sex	-0.184	0.577	37.9
SD (Mother)	1.538	0.920	NA
SD (Year)	0.701	0.550	NA

Table C. Results from the GLMM testing for the influence of infant presence and inter-conception interval on fetal loss probability. This model ran on 166 pregnancies of 52 females, including 28 that resulted in a fetal loss. Standard deviations (SD) of random effects indicate the degree of variation attributable to each grouping factor. $R^2 = 0.30 \pm 0.07$ (estimate $\pm$ error).

Response variable: Fetal loss probability

Parameter	Estimate	Est.Error	Positive draws (%)
Intercept	-3.160	0.803	0.0
Female kin	-0.020	0.146	44.9
Alpha male takeovers	0.566	0.308	97.0
Mother's age	-0.457	1.000	32.1
Mother's age^2	0.277	0.994	60.6
Mother's rank	-0.831	0.317	0.4
|Days to conception peak|	0.370	0.277	91.1
Foraging rate	0.034	0.286	54.0
Inter-conception interval	0.100	0.939	53.7
Inter-conception interval^2	-0.508	0.975	30.4
Infant presence	1.884	0.555	100.0
SD (Mother)	0.627	0.470	NA
SD (Year)	1.235	0.681	NA

Table D. Results from the GLMM testing for the influence of the time since having an infant on fetal loss probability. This model ran on 202 pregnancies of 62 females, including 30 that resulted in a fetal loss. Standard deviations (SD) of random effects indicate the degree of variation attributable to each grouping factor. $R^2 = 0.33 \pm 0.08$ (estimate $\pm$ error).

Response variable: Fetal loss probability

Parameter	Estimate	Est.Error	Positive draws (%)
Intercept	-2.613	0.979	0.3
Female kin	0.017	0.176	53.9
Alpha male takeovers	0.406	0.341	88.6
Mother's age	-4.781	5.500	17.3
Mother's age^2	0.617	4.900	56.2
Mother's rank	-1.115	0.441	0.1
|Days to conception peak|	0.529	0.343	96.0
Foraging rate	-0.056	0.326	43.0
Time since having infant	-19.163	9.335	0.9
Time since having infant^2	10.836	6.966	95.6
SD (Mother)	1.211	0.909	NA
SD (Year)	1.531	0.817	NA

Table E. Results from the GLMM testing for the influence of infant age and mother's pregnancy status on the probability of nursing. This model ran on 8819 focal observations of 314 infants. Standard deviations (SD) of random effects indicate the degree of variation attributable to each grouping factor. $R^2 = 0.15 \pm 0.01$ (estimate $\pm$ error).

Response variable: Nursing probability

Parameter	Estimate	Est.Error	Positive draws (%)
Intercept	-5.696	0.384	0.0
Pregnancy status	0.417	0.620	74.7
Infant age	-0.003	0.001	0.1
Infant sex	-0.004	0.124	47.9
Mother age	-0.031	0.015	1.7
Mother rank	0.214	0.339	73.8
Pregnancy:Inf.age	-0.002	0.002	21.0
SD (Infant)	0.528	0.080	NA
SD (Mother)	0.212	0.105	NA
SD (Year)	0.355	0.169	NA

Table F. Results from the GLMM testing for the influence of infant age and mother's pregnancy status on the probability of carrying. This model ran on 8819 focal observations of 314 infants. Standard deviations (SD) of random effects indicate the degree of variation attributable to each grouping factor. $R^2 = 0.30 \pm 0.01$ (estimate $\pm$ error).

Response variable: Carrying probability

Parameter	Estimate	Est.Error	Positive draws (%)
Intercept	-3.832	0.668	0.0
Pregnancy status	-0.565	0.733	21.6
Infant age	-0.014	0.001	0.0
Infant sex	-0.175	0.183	16.7
Mother age	-0.009	0.023	35.1
Mother rank	-0.145	0.497	38.6
Pregnancy:Inf.age	0.002	0.003	81.9
SD (Infant)	0.574	0.150	NA
SD (Mother)	0.615	0.143	NA
SD (Year)	1.189	0.377	NA

Table G. Results from the GLMM testing for the influence of infant age and mother's pregnancy status on the probability of tantrum. This model ran on 8819 focal observations of 314 infants. Standard deviations (SD) of random effects indicate the degree of variation attributable to each grouping factor. $R^2 = 0.07 \pm 0.01$ (estimate ± error).

Response variable: Tantrum probability

Parameter	Estimate	Est.Error	Positive draws (%)
Intercept	-7.210	0.708	0.0
Pregnancy status	-0.379	0.813	31.3
Infant age	-0.005	0.002	0.2
Infant sex	0.108	0.177	73.3
Mother age	-0.032	0.021	6.6
Mother rank	-0.235	0.457	30.0
Pregnancy:Inf.age	0.002	0.003	78.2
SD (Infant)	0.212	0.151	NA
SD (Mother)	0.188	0.130	NA
SD (Year)	1.113	0.396	NA

Table H. Results from the GLMM testing for the influence of infant age and mother's pregnancy status on the probability of rejection. This model ran on 8819 focal observations of 314 infants. Standard deviations (SD) of random effects indicate the degree of variation attributable to each grouping factor. $R^2 = 0.03 \pm 0.01$ (estimate ± error).

Response variable: Rejection probability

Parameter	Estimate	Est.Error	Positive draws (%)
Intercept	-8.958	0.603	0.0
Pregnancy status	1.785	0.761	98.8
Infant age	0.002	0.002	87.7
Infant sex	-0.294	0.169	4.0
Mother age	-0.032	0.020	5.3
Mother rank	0.361	0.436	79.8
Pregnancy:Inf.age	-0.005	0.003	3.2
SD (Infant)	0.162	0.117	NA
SD (Mother)	0.131	0.097	NA
SD (Year)	0.504	0.216	NA

Table I. Results from the GLMM testing for the influence of mother-infant interaction rates on fetal loss probability. This model ran on 89 pregnancies of 40 females, including 22 that resulted in a fetal loss. Standard deviations (SD) of random effects indicate the degree of variation attributable to each grouping factor. $R^2 = 0.37 \pm 0.09$ (estimate $\pm$ error).

Response variable: Fetal loss probability

Parameter	Estimate	Est.Error	Positive draws (%)
Intercept	-1.035	0.853	10.0
Female kin	0.267	0.399	75.2
Mother's rank	-1.342	0.938	8.0
Mother's age	-0.115	0.997	45.4
Mother's age^2	0.276	0.958	60.2
|Days to conception peak|	0.267	0.400	75.2
Alpha male takeovers	0.978	0.380	99.8
Foraging rate	0.264	0.402	75.5
Time from birth to conception	0.549	0.399	92.4
Tantrum rate	0.680	0.392	96.2
Carrying rate	0.240	0.485	70.7
Nursing rate	-0.186	0.459	33.0
Rejection rate	-0.438	0.405	13.9
SD (Mother)	1.407	0.959	NA
SD (Year)	0.614	0.583	NA

Table J. Results from the GLMM testing for the influence of male sexual coercion (intimidation) to the female on fetal loss probability. This model ran on 255 pregnancies of 97 females, including 43 that resulted in a fetal loss. Standard deviations (SD) of random effects indicate the degree of variation attributable to each grouping factor. $R^2 = 0.29 \pm 0.06$ (estimate $\pm$ error).

Response variable: Fetal loss probability

Parameter	Estimate	Est.Error	Positive draws (%)
Intercept	-2.507	0.541	0.0
Female kin	0.045	0.229	59.0
Alpha male takeovers	0.412	0.262	94.4
Coercion rate	-0.217	0.440	34.2
Mother's age	-0.310	0.971	37.5
Mother's age^2	0.187	0.980	57.9
Mother's rank	-0.469	0.232	1.9
|Days to conception peak|	1.077	0.315	100.0
Foraging rate	0.172	0.246	75.5
Infant presence	1.322	0.483	99.7
SD (Mother)	0.413	0.325	NA
SD (Year)	1.074	0.539	NA
