# Supplementary material for: Infant presence and mother-infant conflict predict fetal loss in wild mandrills
Source: PLoS Biol. 2026 Nov 3;24(11):e3003970. doi: 10.1371/journal.pbio.3003970 (PMC13730340; doi:10.1371/journal.pbio.3003970)
Supplement: Supplementary file 2 [file pbio.3003970.s005.pdf]

Turku, May 27th 2026

Dear Editorial office,

We thank you and the three reviewers for providing useful comments for our manuscript entitled "Infant presence and behavior predict maternal fetal loss in wild mandrills" (updated title). We have modified our manuscript according to these comments and our responses and justifications are detailed in the text below (in red, following each comment).

Please let us know if you require any further information and thank you again for your consideration.

Regards,
N.S.

PS. Please note that we do not upload a revised manuscript with track changes because we work in a dual mode between doc and latex. Apologizes for the inconvenience.

Reviewer #1:

Thank you for the opportunity to read this exciting work! This was an interesting study in a system that permits a rare insight into these dynamics of fetal loss, without necessitating physiological sampling. As a short report, this provides a unique contribution to the literature with parallels to several (scant) works on the Bruce effect, and spontaneous fetal loss from other primates. Generally, the article is clearly written and compelling. I have numerous comments mostly stemming from an interest in this work, or a perceived need for clarity. I do have a slight concern regarding the heaviness of the "adaptive" interpretation and fitness analyses and the mother-offspring conflict, which seem to be overly emphasized given the evidence here (as I understand it). While the statistical analyses are generally reasonable, there are a few nuances that I would benefit from clarity on.
Thank you for your generally positive assessment and for this general comment. We have toned down the adaptive interpretation and deterministic language throughout the manuscript, and we have also removed "adaptive" from the title that now reads "Infant presence and behavior predict maternal fetal loss in wild mandrills".

There are other aspects of this system that I would be curious about: nutritional quality of the surrounding environment during fetal loss (NDVI, rainfall, or direct measures), female health during fetal loss (female condition, rank changes, etc.), male behavior during these periods (alpha male turnover, any tendencies towards infanticide?), rearing of the females themselves (captive versus wild reared; as rearing condition can alter maternal behavior). All of these could alter this system in ways that might influence fetal development, loss, and the general costs of reproduction. As the authors note for some of these, tantrums could be linked to maternal investment or condition. If the authors do not have some of these variables, then perhaps add a brief discussion of their absence as a limitation and future direction for alternative explanations.
Just an elaboration: though the article is focused on mother-offspring dynamics, the male behavior during these periods seems of direct relevance. This has relevance because infanticide has been reported in (captive) mandrills (Setchell et al. 2008) and my impression is that the Bruce effect has been invoked as a preventative for infanticidal strategies by new males (Roberts et al. 2012). The authors briefly explore coercion, but I have a few other questions: Was the alpha male stable across

the study? Did fetal loss coincide with major changes in resident males? Even qualitative insights into these questions would be of interest to the target audience.
Nutritional quality and health: we have now added in our models the foraging rate of the female during the first month of pregnancy, as a proxy of food availability. Relevant results are now included in the paragraph “Fetal loss and infant presence” of the supplementary material: “The number of maternal kin, female age, and foraging rate have a weak effect on fetal loss probability in all three models (…). ”

Rank changes: we control for female rank in our analyses but note that female ranks are generally stable (and maternally inherited) in mandrills.

Captive- vs wild-born females: from the 117 females, only 16 were born and raised in captivity. We have now run our analysis including only wild-born females and the results are, again, qualitatively similar (see the revised paragraph “Fetal loss and infant presence” of the supplementary material).

Male-mediated effects: We have now added in our models the number of alpha male takeovers during the first month of pregnancy. We used data on the first month of pregnancy to standardize comparisons (because all pregnancies last at least a month). Fetal loss probability is greater for pregnancies associated with more alpha male takeovers. We now present this result in the paragraph “Fetal loss and reproductive success” and we also added a figure (Fig 3) to illustrate it.

I am not wholly convinced that there is conflict here. The survival of the infant seems to be advantageous to both the mother and the infant. Lactation is, of course, the costlier process (Emery Thompson, 2024) and has already been invested into the living infant. Assuming mothers cannot support both offspring, then fetal loss is of mutual benefit for both mother and infant; yes? Though, the fetus has a conflicting priority at this stage - so, perhaps this is really inter-offspring conflict? Finally, there is a good deal of literature on the fetus' role in securing its own survival (e.g., Fowden & Moore, 2012; Sterner et al. 2012), which seems worth discussing and citing of the relevant works.
We thank you for this insightful comment and agree that infant survival may be beneficial for both mothers and their dependent infants. However, females producing two surviving offspring within a short time window appear to achieve the highest medium-term reproductive success (Figure 4) and it is intuitively more beneficial for a female to produce two infants. In contrast, an offspring’s interest may be to maximize the maternal resources it receives itself. We therefore interpret our findings within the framework of parent-offspring conflict theory, which describes evolutionary divergence in optimal investment between parents and their offspring. In this context, the evolutionary interests of offspring are aligned with higher levels of maternal investment whereas mothers face allocation trade-offs among current and future offspring (including both fetuses and dependent infants). That being said, inter-offspring conflict (“sibling competition”) is also relevant to our study. We have thus now included this notion in our revised manuscript (first paragraph of the introduction L15 – where we also cite the suggested literature).

Finally, these rates of fetal loss seem well within the range of what we expect from all primates. If this is true, then this 'adaptive' fetal loss falls in the same range as 'general' (for lack of a better distinction) fetal loss. If this was adaptive for mandrills, why is the rate fairly consistent with what we see across primates? (see my comment to L168)
The "true" rate of fetal loss is probably higher than the one reported as our field methods do not allow to detect early fetal losses. Yet, even if the rate of fetal loss in mandrills is similar to those observed in other primates, our results may highlight that the mechanism we report is common and presumably adaptive in other primates too. Please see our response to your comment on ex-L168 for more details.

Section and Line Comments:
The title unambiguously defines fetal loss as adaptive, but the abstract presents resource allocation constraints as a (non-mutually exclusive) alternative. I would prefer adaptive removed from the title, if it is ambiguous throughout. The Teaser is more appropriately couched in fitness outcomes (rather than an adaptive strategy)
We have now changed our title to better reflect our results and avoid an adaptive perspective. We have further clarified that fetal loss in response to resource constraints may also be adaptive (e.g., L105). Finally, we have also toned down any deterministic language in our revised manuscript.

Figure 1 is presented while the study is still being set up to the reader, yet is presenting study outcomes before they have been addressed in the text. I recognize this work is a short report, but this structural position was confusing.
Figure 1 represents an introductory figure explaining the setup of our study and our central hypothesis. We have now added in the figure legend that it presents "hypothesized pregnancy outcomes". We have placed it after the study is introduced but before the results, so it visually helps the reader to understand the setup.

Figure 2: If I understand correctly and all of these lines are plotted from the model posteriors, then add uncertainty to the plots. If they are not from model posteriors, then please re-plot them using model posteriors (and add uncertainty).
We have now added uncertainties to our plots and specified in the legend that we use model posteriors to generate them.

Figure 2c: I do not recall reading a rationale for the lower number of tantrums in infants of pregnant females? This would be beneficial to explicitly resolve or hypothesize about. Also, would it be better to know the x-axis relative to conception age or day until expected birth?
We have updated this analysis using data only on infants older than 6 months because we do not really have observations of younger infants of pregnant mothers, and this is the reason that we previously saw this effect. Now, tantrum probability declines with infant age for both pregnant and non-pregnant females and while it is generally lower for non-pregnant females (see attached Figure), the difference is not statistically robust. We did the same (using only data on infants at least six months old) also for our models considering nursing, carrying, and maternal rejection (see main text L349).

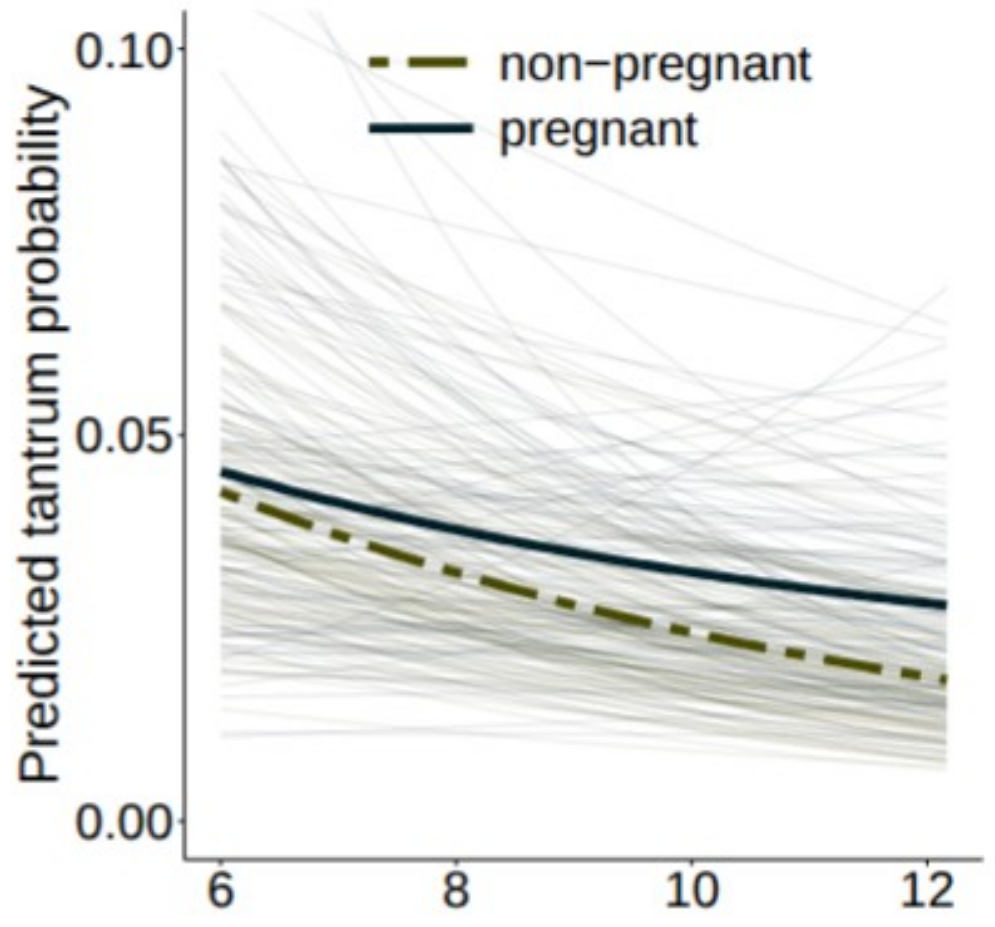

Our analysis considers pregnancy as a binary variable (pregnant vs non-pregnant) and in interaction with infant age, therefore, we think it is more accurate to illustrate the effects with infant age in the x-axis. Using conception dates would result in comparisons of infants of very different ages and infant age is a strong predictor of mother-infant interactions.

L126-132: During what period were the tantrum rates calculated? Note that I read through the relevant Methods section and this remained unclear to me.
We have now changed to "We then studied whether greater maternal care and/or intensified mother-infant behavioral conflict are associated with a greater probability of fetal loss (response variable 0/1), using a GLMM with a binomial distribution and a logit link function, and controlling for infant age and other relevant factors (care and conflict rates were calculated during the infant's first year of life, see methods; Table S9)". This is the default 'infant period' across our study.

Figure 3 & 143-161: Nuance is missing here in that females are unlikely to have only 2 offspring during their lifespan. It would be useful to know this all in the context of lifetime reproduction. The strategy of having multiple consecutive offspring seems more optimal in high quality circumstances, as supported by your findings of high ranking females practicing this strategy. If consecutive reproduction is successful, what is the benefit for lifetime reproductive output? If females are only having a lifetime output of 6-10 offspring, for instance, then the opportunity to have 3 infants in the span of 4 years (two consecutive, and one as females return to an 'every other season' cycle) is a big potential boon relative to a female sticking to the every-other-season strategy (which will yield 2 infants). The outcome would still be 2 infants were she to lose her middle infant, and still high (as you note in Figure 3) with fetal loss and rapid re-fertilization (3 infants). Some nuanced discussion of this is warranted given females have more than 2 offspring in their lives (I assume). More concisely, what are the lifetime fitness implications for females that: never have consecutive births, always have consecutive births with survival, or have a mixed strategy. Then present these same combinations but with fetal loss and infant death complications for lifetime fitness.
It is not straightforward to compare lifetime reproductive success of females who experience fetal loss and those who have consecutive successful births as the pace of female reproduction may be influenced by factors other than fetal loss (e.g., dominance rank). Therefore, females that reproduce without experiencing fetal losses or infant deaths between successful births might still show variable IBIs. Additionally, a female might have one, two or more fetal losses during her lifetime. Therefore, we cannot easily classify females to the proposed categories (never had consecutive births, always had consecutive births with infants surviving after 12 months, or a mixed strategy).

The maximum female lifespan observed in this population is 24 years representing ~20 year of reproductive lifespan. Our study is based on 11 yrs of reproductive history failing to quantitatively assess lifetime reproductive success in the study females. However, we have now added in our discussion the following information that partially addresses this comment: "Thus, fetal loss could be a key determinant of reproductive success, partly contributing to the large variation in lifetime reproductive success observed among females in this population (12 females who were alive and of reproductive age throughout the 11-year study period produced between 2 and 9 living offspring)" (L195).

L168: The authors describe fetal loss as occurring in 10-20% of pregnancies across many primates. Given the observed fetal loss in mandrills is well within that range (52/356 * 100 = 14.6%), why do we need any explanatory variables here? That is, how does this circumstance differ from other primate systems? Why are these rates higher than we might expect from average rates attributable to random mutation or developmental complications? If they are not, then is there any reason to believe infants are not just cuing behavior off of the mother's behavior/physiology?
Thank you for this important point. First, as stated above, the fetal loss rate in our study population is likely underestimated, as early pregnancy terminations are not detected. Second, even if the overall rate of fetal loss in mandrills falls within the range reported in other primates, our analyses highlight that the mechanism we report (i.e., infant presence as a driver of fetal loss) may be common in other primates too. This suggests that, beyond "baseline" causes such as developmental

anomalies or random mutational processes, social and life-history factors may contribute to shaping fetal loss probability. These baseline causes likely still operate, but our data do not allow us to disentangle their contribution. Addressing these mechanisms would require different types of data and falls beyond the scope of the present study.
Finally, and if interpreting correctly the last sentence of this comment, i.e., that fetal loss and infant tantrums have a common cause (e.g., poor female condition), we have now added that interpretation in our revised text to address this comment: "This result may reflect two non-exclusive mechanisms: either variation in maternal condition contributes to both greater tantrum rates and increased fetal loss probability, although in this case we would also expect changes in other proxies of maternal care, or infant behavior itself contributes to variation in fetal loss probability. The latter mechanism would represent an infant strategy delaying the birth of a younger sibling to prolong the period of exclusive maternal investment. Similar strategies have been described in other species, where offspring interfere with maternal sexual activity (..) or chemically suppress parental reproduction delay parental reproduction through physiological pathways (..). Disentangling these mechanisms would require detailed data on maternal condition, reproductive physiology and behavioral interactions."

L174: I feel the need for some resolution, somewhere in the article, why there is a conflict? Assuming insufficient resources to support the fetus and lactation, then there is no conflict - because the sunk-cost in the living infant is of greater priority for the mother as well?
We agree that when maternal resources are insufficient to sustain both gestation and lactation, fetal loss may be consistent with the mother's immediate energetic constraints. However, parent–offspring conflict does not arise solely from a trade-off between a current fetus and a dependent infant, but from differences in optimal levels of maternal investment from the perspective of the mother and her offspring. In this context, even when the mother's optimal strategy involves prioritizing the dependent infant over the fetus, conflict may still arise over the amount and duration of investment allocated to the infant itself. Dependent infants are expected to request higher levels of maternal investment than those that maximize maternal lifetime reproductive success, particularly when mothers face constraints on future reproduction. More broadly, short inter-birth intervals place mothers in a situation where investment must be allocated between current and future reproduction, and conflict may emerge over both the timing of reproduction and the distribution of resources across offspring. Our results suggest that such conflicts may be exacerbated when gestation and infant care overlap. We have now edited our introductory paragraphs to clarify the presence of conflict (e.g., L18).

L189: I would value some elucidation of whether any of these fetal loss patterns were biased towards captive- or wild-reared mothers. I recognize that this would co-vary with age.
Please see our response above and the results in the supplementary material. When we ran our main model including only wild-born females, the results remained very similar.

L281: Please describe "qualitatively similar". Did the meaningful effects remain?
We now specify that "when we excluded from our models the highest-ranking females (with standardized Elo-rating>0.8), our results remained similar: pregnant females with a dependent infant showed a greater probability of fetal loss than those without, with a 100% posterior probability of a positive effect" (L327).

Statistical Analyses
There is little mention of scaling variables (though I see, for instance, rank is standardized). Scaling is especially important when including binary and continuous variables (Gelman, 2007). Please confirm that continuous variables were scaled (and rerun analyses, if not, to confirm results are the same).

We have now specified in the last paragraph of the methods (L410) that "We used function *scale* from base R to standardize continuous variables fit as predictors in the GLMMs".

Please report outcomes of posterior predictive checks and screening for multicollinearity (pp_check() and pairs() plots in brms)
We have now specified in the last paragraph of the methods (L413): "We assessed model fit using posterior predictive checks with functions/plots *pairs* and *pp_check* from package *brms* […] We used function *correlationBF* of package *BayesFactor* to calculate correlation coefficients and assess multicollinearities".

How do you discriminate between meaningful and non-meaningful effects? Are you relying on a 95% positive draw cut-off? Two-tailed or one-tailed?
We purposely avoid using terms as "meaningful" or "significant". We think that using such terms or cut-offs are typical of a frequentist approach. We thus avoid them in a Bayesian context (although other authors report, for example, 89% CIs, to highlight the arbitrariness of such cut-offs). Yet, to improve readability, we have now used expressions like "weakly correlated" while always reporting posterior probabilities.

Are your posterior draws based on the conditional or marginal posterior? Do these estimates and errors include variance from your random effects?
Given that our main questions are population-level based and that we do not focus on random effects, our posterior draws are based on the conditional posteriors (which are also more interpretable). These estimates and standard errors do not include variance from the random effects (Mother and Year); they represent population-level fixed effect estimates conditional on the estimated random effect structure. We have now clarified this point in our revised manuscript (L415).

L325: Please cite R.
Done.

L461: Please cite Biorender in the captions of the relevant figures.
We have removed all Biorender icons and using our own.

Supplementary
Several references are not populated or, rather, populated as: (?)
We apologize for these errors, they are now corrected.

Please report correlation coefficients between Mother age, Infant age, and Mother rank. Also, did you screen for multicollinearity?
We have tested for multicollinearities between the predictors in all our models and have now reported correlation coefficients, as suggested (L319).

Please add, to all the statistical supplementary tables: lower/upper CIs and your meaningful cut-off for % Positive Draws. Also, add random effects and report Bayesian R2 estimates
Please, see our response to a relevant comment above, regarding the usage of cut-offs.
The percentage of positive/negative draws does not depend on any cut-off, but rather on the posterior distribution itself. We have now added random effects in all our tables and Bayesian R2 estimates and their errors in the legends of these tables.

Reviewer #2:

The authors present an interesting study on fetal loss in wild mandrills. In this study, pregnancy and fetal loss were estimated by visually assessing the swellings of females. Using 11 years of data on 117 females (356 pregnancies), the authors tested what factors predict fetal loss and whether fetal loss is evolutionarily beneficial in certain contexts. The analyses suggest that pregnant females with a dependent infant face a higher risk of fetal loss than pregnant females without a dependent infant. Among pregnant females with dependent infants, higher rates of infant tantrums predicted a higher risk of fetal loss. Furthermore, the reproductive success of females who experience fetal loss was better than females who birthed an infant who then soon died. Together the authors posit that fetal loss is a strategy to maximize reproductive success in the context of a too-rapid reproductive pace. I am glad I said yes to this review because I enjoyed reading this report! I think the study will be a nice addition to the literature on female reproduction, maternal-offspring conflict, and life history. There are a few aspects of the manuscript that could be clarified or improved.
Thank you for this positive general comment!

First, infant presence vs recovery time.
"Infant presence, not limited recovery time, increases fetal loss" is the title of the first section. This may not be the most accurate way to frame the results. The analyses show that time since last gestation does not strongly predict fetal loss, but the presence of a dependent infant and time since presence of a dependent infant do strongly predict fetal loss. Since maternal care (lactation, carrying) is so costly, it seems odd to frame the findings this way. Recover time probably does matter; it's just that the mother has not even started recovery yet if there is still a dependent infant! Similarly, there is a dichotomy presented at times, for example in the abstract: "fetal loss may reflect constraints on resource allocation or an adaptive adjustment to the high costs of short inter-birth intervals." While there are different perspectives, many would view fetal loss in response to resource constraints as a potentially adaptive response. If a pregnant female has limited resources, it might be most adaptive to terminate the pregnancy rather than invest the time and energy into a pregnancy that could lead to an infant that does not survive. Under the circumstances of resource constraints, this response would be adaptive. Perhaps some slight rewording could work: fetal loss may reflect an adaptive adjustment to constraints on resource allocation and/or the high costs of short IBIs.
We have changed the title of this section to “Fetal loss and infant presence”.
Fetal loss in response to resource constraints can be, indeed, an adaptive response. This is a valuable perspective, thank you! We have rephrased our abstract as suggested and also edited the text accordingly (e.g., L65).

Medium-term reproductive success.
The analysis to test proxies for fitness could be clarified a bit and/or improved. I understand the decision to use 'a period of two median interbirth intervals' as the unit of measurement, but I found it challenging to read and understand this throughout the paper. How many years is this? It might be easier on the reader to mention the time period instead (and justify the time period by saying it represents two median IBIs).
We should have indeed expressed the length of two median inter-birth intervals in years. Wherever we refer to the two median inter-birth intervals, we have now added the associated length (3.1 yrs).

The window between first conception and next conception must vary across the 3 categories. Maybe this variation could be illustrated in Figure 3? Is the Bayesian t-test the best way to handle this analysis? The outcome variable seems to be a count (# of surviving infants in the time period), so would a Poisson be feasible? The main predictor of interest could be a categorical: fetal loss,

next infant died, next infant survived. I also wonder if the authors considered using IBI as the outcome (similar to the cited Bruce effect paper Roberts et al). For an IBI analysis, the predictor categories of interest could be: two consecutive successful births, fetal loss between successful births/surviving infants, infant death between surviving infants.
The first part of Figure 3 is descriptive and we think that if we add conception, birth and weaning times at different time points on the three axes would worsen the readability of an already rich figure.
We chose a simple, non-multivariate analysis, to compare average medium-term reproductive success. We followed a similar approach to Roberts et al 2012 (on the Bruce effect in geladas), essentially because our sample sizes are restricted to sustain a multivariate analysis (only 8 Born-Died, and only 19 Fetal Loss cases; see right panel of Figure 3).
As suggested, we have now added an alternative analysis to our comparison on the number of infants born within two average IBIs. Specifically, we compare the IBIs between two successful reproductive events (surviving infants) of females that experience a fetal loss, an infant death or no disruption between these two successful reproductions. We have added this new analysis in the last section of our main text (L184), and a relevant supplementary Figure (S2).

Finally, I have a clarifying question about what the authors think is happening here! Is conceiving early just a mistake? Or do some females employ a fast reproductive strategy that works out often enough that it's adaptive on average (i.e., successful pregnancy and surviving infant sometimes) and mistakes can be somewhat mitigated by fetal loss if the pace is too fast at times? This is how I interpreted the conflict: mothers try to reject a lot when pregnant, but if the infant is exhibiting a lot of tantrums, then fetal loss may occur. So, this represents conflict and a negotiation. I do wonder if the tantrums are high and the mother does not experience fetal loss, what is the risk of infant death? (I'm not looking for the authors to provide an answer to this last question, just a thought for context and discussion)
Thank you for this insightful comment. While we know from other species that short IBIs can have detrimental consequences for infant survival (e.g., rhesus macaques) and growth (e.g., chimpanzees), there has been no direct test of the link between the older offspring's behavioral 'efforts' to prevent investment in a new fetus or a new infant and the consequences when such efforts fail.
We do think that conceiving early represents a "trial and error/failure" strategy where females attempt to maximize their reproductive output, occasionally succeeding and sometimes failing. Hence, your interpretation is in line with our view. We have now added in our introduction the following: "Therefore, a female who conceives soon after giving birth faces concurrent allocation demands from both infant and fetus, giving rise to interacting physiological and behavioral conflicts among the female, her living infant, and her fetus, which may influence the survival of both offspring (...). These conflicts may be particularly intensified when conception occurs before weaning of the living infant, as mothers reduce investment and infants continue to solicit high levels care." (L16).

Note: There are a number of "?"s in the supplementary materials
We apologize for these typos, everything has now been corrected.

Reviewer #3:

Smit et al. investigate the evolutionary and behavioral drivers of fetal loss in a wild population of mandrills. Using eleven years of data, including 356 pregnancies across 117 females, the authors conduct a strong empirical test the "adaptive fetal loss" hypothesis. They show that the presence of a dependent infant (under one year old) substantially increases the probability of fetal loss, and that females who experience fetal loss achieve higher medium-term reproductive success than those who carry an infant to term only for it to die shortly after birth.

I thoroughly enjoyed this interesting, well-written, and important manuscript and consider it the best paper I have reviewed in at least a year (for reference, I review about 25 manuscripts per year and edit roughly the same number). The study system (daily monitoring for 11 years, the ability to reliably identify cycling, pregnancy, and fetal loss) provides a lovely opportunity to test ideas central to life history theory and maternal-offspring conflict.

Analyses: I found the statistical analyses to be appropriate, clearly and succinctly explained, and transparently reported (e.g., Table S1-S10). The Bayesian GLMM framework allows for a nuanced understanding of the posterior probabilities of various drivers (reported as continuous values reflecting the percent positive posterior probability), rather than dichotomously characterizing effects as significant or non-significant.

Figures: Each of the three figures are effective, especially figure 1 that clearly depicts the conceptual timeline of female mandrill reproduction and figure 3, which is an outstanding visual depiction of the main results of the paper. I generally dislike the "graphical abstracts" sent out with email tables of contents from journals, in part because most of the figures are not that effective. If most graphical abstracts were as clear and effective as Smit et al.'s figure 3 I would feel differently. Very well done.
Thank you very much for these very positive comments! We appreciate them!

I see three areas where the authors could improve on the manuscript.

1. Overall, the authors were circumspect about the interpretation of their results and were admirably careful to not oversell their findings- with one exception. I found the causal language regarding the effect of mother-infant behavioral conflict to be too strong. For example, lines 18-19 stated that "behavioral conflict… triggers fetal loss", but this is supported by a correlation- there are no results (e.g., from non-invasively measured hormones) that causally link fetal loss through some stress-induced physiological pathway. I suggest the authors rein in their framing here.
We have now toned down our text as suggested. In the abstract, we now write “females who conceive while still caring for a young infant experience both a greater probability of fetal loss and increased mother–infant behavioral conflict in the form of maternal rejection”. Additionally, we have changed the titles of all sections to be more descriptive and not imply causality.

2. There are unmeasured factors that could potentially confound the results. For example, while maternal rank and age are included as covariates, it is possible that maternal health could play a role here, too. Similarly, the random effect for year does not "control for" environmental confounds that might make years vary in quality. While the strength of the detected effects using the data presented reassure me that the results are likely robust, finding a way to assess the impacts of i) maternal health and ii) environmental quality (e.g., using phenology or rainfall data) would further strengthen the results and increase confidence in the conclusions.

We have now added in our models the foraging rate of the female during the first month of pregnancy, as a proxy of food availability and potentially female condition. Relevant results are now included in the paragraph “Fetal loss and infant presence” of the supplementary material: “The number of maternal kin, female age, and foraging rate have a weak effect on fetal loss probability in all three models.”

3. As the authors transparently admit, pregnancy was detected via swelling, which occurs several weeks post-conception. This means the "true" rate of fetal loss is likely higher that they were able to detect. While this is an inherent limitation, I would have appreciated consideration of how this systematic undercounting might affect the results and interpretation.
We, indeed, probably miss some early cases of fetal loss. We think, however, that the strong effect of infant presence we describe is unlikely to be influenced by the lack of information on these early fetal losses (occurring at the very early stages of pregnancy). We further suspect that our effect would be even stronger when considering early fetal loss as dependent infants would be younger. We have now added in the text that “including earlier fetal losses is unlikely to alter our conclusions, as these cases would involve younger dependent infants, a context in which maternal exhaustion or mother–infant conflict may be even more pronounced than in females experiencing fetal loss later.”

Specific points
- Line 33: clarify who you mean by "their"
We have rephrased to use “the” instead.

- Figure 2: would it be possible to include the Bayesian equivalent of confidence intervals, credibility intervals, on the lines in panels c-f?
We have now added uncertainty to our plots.

- Line 167: the value 4.5% is not indicated anywhere in Figure 3. I suggest in these parenthesis the authors indicate that 4.5% is 8 of 176, to tie it more clearly to the figure.
We have added the requested information within parentheses: (8/157=5.1%) (L203).

- When models included random effects the authors noted them as "random factors" (e.g., 261, 294, 311). Please specify what the random effects are. I assume they are random intercepts, but it is possible they could be random slopes, random slopes and intercepts (correlated or uncorrelated), so specifying would be welcome.
We have now specified that we used intercepts, everywhere in the text where we mentioned random factors. We have also added estimates and errors of these random factors in all revised tables.
