# Supplementary material for: Infant presence and mother-infant conflict predict fetal loss in wild mandrills
Source: PLoS Biol. 2026 Nov 3;24(11):e3003970. doi: 10.1371/journal.pbio.3003970 (PMC13730340; doi:10.1371/journal.pbio.3003970)
Supplement: Supplementary file 3 [file pbio.3003970.s006.pdf]

Turku, July 25th 2026

Dear Editorial office,

We thank you and the reviewers for providing additional comments for our manuscript entitled "Infant presence and mother-infant conflict predict fetal loss in wild mandrills" (updated title). We have modified our manuscript according to these comments and our responses and justifications are detailed in the text below (in red, following each comment).

Please let us know if you require any further information and thank you again for your consideration.

Regards,
N.S.

---------------------------------------------------------------------

**Academic Editor:**

a) Overall, the Academic Editor is largely satisfied and has provided some comments for your consideration (below).
Thank you for your consideration.

b) Please address my Data Policy requests below; specifically, you need to supply the underlying data for Figs 2ABCD, 3, 4, S1, S2. The data needs associated data repository, such as Zenodo with a DOI or a supplementary data file.
We have now deposited all relevant code and data in Zenodo (https://doi.org/10.5281/zenodo.21507883).

c) Please clearly cite the location of the data in all relevant main and supplementary figure legends, for example: "The data underlying this figure can be found at doi.org/10.xxx/zenodo.xxxxx." We understand that this may appear repetitive, but it helps make the figures and their legends more self contained.
We have now added this information in all relevant figure legends.

d) Please include the URLs of your funders in the Financial Disclosure statement.
We have added the URLs as requested.

e) Please make any custom code available, either as a supplementary file or as part of your data deposition.
We have uploaded the relevant scripts in the same repository as the data. They include legends of the data tables, the statistical models, as well as the steps for the replication of all figures.

f) In order to make the Title more specific and appealing, please change it to "Infant presence and tantrum behavior predict maternal fetal loss in wild mandrills".
We added "mother-infant conflict" to the title, to make it more specific:Infant presence and mother-infant conflict predict fetal loss in wild mandrills".

**Reviewer #1**:

I want to emphasize my appreciation for the authors taking into consideration my comments from the last review. I recognize that re-running models with new variables can be time consuming. The majority of my questions and comments have been addressed. I do have a few more comments and suggestions, mostly in the presentation of results.
Thank you for this positive comment.

As an aside, I would encourage the authors to generate a compare document with track changes for reviewers in the future. This can be accomplished in many ways that should be compatible with a Latex to doc workflow. Three options come to mind: Overleaf offers a compare function, the authors can compare *.doc files using Word or LibreOffice Writer (which is free), or latexdiff might work. Additionally, please include line numbers for where changes occur (which were only included sometimes). Both of these changes improve the expedience and ease of the review process.
We used latexdiff, as suggested, and we uploaded a pdf with the changes visible (deletions in red and additions in blue).
We have now included line numbers in all relevant responses. These lines numbers are based on the pdf file with the changes produced with latexdiff.

I understand the desire to not rely on CIs. Even so, it is still essential to include a statement about how the authors interpret their estimates, posterior draws, and effect sizes. Then be consistent in decision making and terminology. For example, in their response the authors state they "have now used expressions like 'weakly correlated' while always reporting posterior probabilities." How do the authors decide what is 'weak' (e.g., L63, L75, L118)? This comment is also applicable to other phrases, like: "little association" (L131), &: "this difference is not statistically supported (50% posterior probability)" (L184). And similarly throughout, where applicable. Do these descriptors differ from each other? If so please unify them, in addition to providing clear text for how you made your inferences. This is essential for readers to understand the authors' rationale.
We added in the last paragraph of the methods the following sentence: "*We consider an effect to provide little evidence of association when fewer than 89\% of posterior samples share the same sign, that is, when less than 89\% is positive and less than 89\% is negative.*"L438

In the response, the authors state that: "…our posterior draws are based on the conditional posteriors (which are also more interpretable). These estimates and standard errors do not include variance from the random effects". It is my understanding that these are not Conditional effects. I believe the authors use as_draws_df() which, I believe, provides Marginal effects. My understanding is that Marginal effects do not include variance from REs, while Conditional effects do. Regardless, I think it would be best practice to just report both types of posterior draws. Consider using conditional effects for a typical hypothetical group. E.g., something like: posterior_epred(model, … , re_formula = NULL, allow_new_levels = TRUE, sample_new_levels = "uncertainty"). And marginal effects, something like: posterior_epred(model, … , re_formula = NA). Conditional effects are worthwhile to understand whether the findings are robust relative to group-level variance from this population
(not just generalizability).
Indeed, we mixed up "conditional" with "marginal" in our previous response - we apologize for that mistake (we were reporting marginal effects, that is, population-level [fixed] effects, which represent estimates averaged over the random effect structure). We have now corrected the revised manuscript (L434). Conditional effects are not straightforward to report because we need to choose which single point represents "the" conditional effect. It could be the mean of "x", max "x", or even the slope of the curve. Due to this reason, we choose not to present conditional effects.

Maternal rank seemed to be a recurring variable of interest (5/6 models had many [>90%] negative draws - assuming no draws were exactly zero), yet I did not find much discussion of this variable.

Indeed there is no mention of [maternal] rank in the Results/Discussion between lines 42 (Introduction) to 284 (Methods). Given its influence in the models, please add this content to the discussion.
Results related to maternal rank are indeed interesting. Given that the effect of maternal rank on fetal loss probability has been already shown in a previous article on the study population (Dezeure et al 2022), we chose to present the result of maternal rank on fetal loss probability in the supplementary material (first paragraph of results).

Line-by-line comments:

L64: The end of this line is cut-off
We completed the sentence. L65

L122: "Rejection probability in pregnant and non-pregnant females converge…" should be "Rejection probabilities…", yes?
Yes, we corrected as suggested. L133

L122: How do you qualify/quantify convergence?
Thank you for pointing this out. We have revised the text adding a more descriptive statement: ". Notably, the difference in rejection probabilities in pregnant and non-pregnant females decreases as infants approach one year of age (...)." L132

L134: Figure 2c should be Figure 2d, yes?
Yes, we corrected as suggested.

L145-169: This paragraph had some odd transitions. The work starts with female-female competition, only really discusses male takeovers. Then, it feels like the new sentence on L163 starts a different paragraph. Please revise this section for clarity. My recommendation would be that L144-163 would work better as its own subsection.
In this paragraph, we first introduce the idea that "*female mammal physiology can indeed, to some extent, influence pregnancy onset or termination in ways that mitigate fitness costs*". We support this claim by citing a study on humans and providing references on the Bruce effect observed in geladas and on the female-female competition effect on fetal loss observed in banded mongooses. However, we do not claim that we do test for all these effects. We have now rephrased the paragraph to improve clarity. Finally, we have also split the paragraph as suggested. L163-176

L153: The authors bring up female-female competition, but it is unclear what variables assesses that. I might assume that it is maternal rank? If so, then that finding can be intuitively discussed here. Instead, only male takeovers is discussed. My supposition is that both are important, but I would value the authors' explicit insights.
We did not explicitly test for female-female competition but we referred to it to support the argument that female physiology can, to some extent, influence pregnancy termination. Testing such effect would require other data than maternal rank and we feel it is beyond the scope of this study. We hope that the revised paragraph is clearer. L167

L193-194: Please add precision as to what timeframes you mean when stating: "short-term" and "medium-term"?
We have now rephrased as follows: *Altogether, these results suggest that fetal loss, while costly in the short-term (i.e., during a given reproductive event), may mitigate medium-term fitness consequences (i.e., over a period including two consequent reproductive events) arising when females are physiologically unable to sustain short inter-birth intervals.* L196

L204: I think a Conclusion subheading would make the transition from the preceding text less awkward.
We added a *Conclusion* subheading as suggested. L218

L204-222: Adding your alpha male takeovers finding might connect this work more completely with relevant literature.
While we agree that this is a very interesting finding that links our study to the broader literature regarding the determinants of fetal loss, we think that it is a "secondary" finding of our analyses. Our study did not focus on male takeovers and male infanticide (neither methodologically, nor conceptually) and thus, we prefer to keep this last paragraph focused on the more original results regarding the effects of infants on maternal fetal loss.

L310-329: I found the presentation of rho with the posterior draws confusing given only the Bayesian GLMM has been discussed here. Traditionally and in the rest of your manuscript, the estimate is presented with posterior draws. I suggest making the Spearman's correlations distinct from the posterior draws, or clarify what is going on in this passage if I have misinterpreted.
Using "rho" was indeed confusing. In fact, we used Bayesian correlation tests (as we note at the end of the methods: we used function *correlationBF* of package *BayesFactor*), and we have now replaced "mean rho" with "mean correlation coefficient" in all relevant points in the text, to avoid confusion.

L412: What was the number of warm-up iterations and the value for thin?
We have now added this information: *We used informative priors (normal (0,1)) for continuous variables and four chains with 2000 iterations in each. The first 1000 iterations were discarded as warmup, retaining 4000 post-warmup draws with no thinning.* L430

R & its packages: Please add version numbers for the version of R you used, and the packages. Or, confirm that these details are clear in your code/readmes.
We added version numbers in the last paragraph of the methods where we present the R packages used. L426-441

The Supplementary Materials still has content populated as: [?]. Especially in the Supplementary Analysis on Sexual Coercion.
We apologize for these typos that have now been corrected.
