# Supplementary material for: Infant presence and mother-infant conflict predict fetal loss in wild mandrills
Source: PLoS Biol. 2026 Nov 3;24(11):e3003970. doi: 10.1371/journal.pbio.3003970 (PMC13730340; doi:10.1371/journal.pbio.3003970)
Supplement: Supplementary file 4 [file pbio.3003970.s004.docx]

I want to emphasize my appreciation for the authors taking into consideration my comments from the last review. I recognize that re-running models with new variables can be time consuming. The majority of my questions and comments have been addressed. I do have a few more comments and suggestions, mostly in the presentation of results.

As an aside, I would encourage the authors to generate a compare document with track changes for reviewers in the future. This can be accomplished in many ways that should be compatible with a Latex to doc workflow. Three options come to mind: Overleaf offers a compare function, the authors can compare *.doc files using Word or LibreOffice Writer (which is free), or latexdiff might work. Additionally, please include line numbers for where changes occur (which were only included sometimes). Both of these changes improve the expedience and ease of the review process.

I understand the desire to not rely on CIs. Even so, it is still essential to include a statement about how the authors interpret their estimates, posterior draws, and effect sizes. Then be consistent in decision making and terminology. For example, in their response the authors state they “have now used expressions like ‘weakly correlated’ while always reporting posterior probabilities.” How do the authors decide what is ‘weak’ (e.g., L63, L75, L118)? This comment is also applicable to other phrases, like: “little association” (L131), &: “this difference is not statistically supported (50% posterior probability)” (L184). And similarly throughout, where applicable. Do these descriptors differ from each other? If so please unify them, in addition to providing clear text for how you made your inferences. This is essential for readers to understand the authors’ rationale.

In the response, the authors state that: “…*our posterior draws are based on the conditional posteriors (which are also more interpretable). These estimates and standard errors do not include variance from the random effects*”. It is my understanding that these are not Conditional effects. I believe the authors use as_draws_df() which, I believe, provides Marginal effects. My understanding is that Marginal effects do not include variance from REs, while Conditional effects do. Regardless, I think it would be best practice to just report both types of posterior draws. Consider using conditional effects for a typical hypothetical group. E.g., something like: posterior_epred(model, … , re_formula = NULL, allow_new_levels = TRUE, sample_new_levels = "uncertainty"). And marginal effects, something like: posterior_epred(model, … , re_formula = NA). Conditional effects are worthwhile to understand whether the findings are robust relative to group-level variance from this population (not just generalizability).

Maternal rank seemed to be a recurring variable of interest (5/6 models had many [>90%] negative draws – assuming no draws were exactly zero), yet I did not find much discussion of this variable. Indeed there is no mention of [maternal] rank in the Results/Discussion between lines 42 (Introduction) to 284 (Methods). Given its influence in the models, please add this content to the discussion.

Line-by-line comments:

L64: The end of this line is cut-off

L122: “Rejection probability in pregnant and non-pregnant females converge…” should be “Rejection probabilities…”, yes?

L122: How do you qualify/quantify convergence?

L134: Figure 2c should be Figure 2d, yes?

L145-169: This paragraph had some odd transitions. The work starts with female-female competition, only really discusses male takeovers. Then, it feels like the new sentence on L163 starts a different paragraph. Please revise this section for clarity. My recommendation would be that L144-163 would work better as its own subsection.

L153: The authors bring up female-female competition, but it is unclear what variables assesses that. I might assume that it is maternal rank? If so, then that finding can be intuitively discussed here. Instead, only male takeovers is discussed. My supposition is that both are important, but I would value the authors’ explicit insights.

L193-194: Please add precision as to what timeframes you mean when stating: “short-term” and “medium-term”?

L204: I think a Conclusion subheading would make the transition from the preceding text less awkward.

L204-222: Adding your alpha male takeovers finding might connect this work more completely with relevant literature.

L310-329: I found the presentation of *rho* with the posterior draws confusing given only the Bayesian GLMM has been discussed here. Traditionally and in the rest of your manuscript, the estimate is presented with posterior draws. I suggest making the Spearman’s correlations distinct from the posterior draws, or clarify what is going on in this passage if I have misinterpreted.

L412: What was the number of warm-up iterations and the value for thin?

R & its packages: Please add version numbers for the version of R you used, and the packages. Or, confirm that these details are clear in your code/readmes.

The Supplementary Materials still has content populated as: [?]. Especially in the Supplementary Analysis on Sexual Coercion.
