# Supplementary material for: Infant presence and mother-infant conflict predict fetal loss in wild mandrills
Source: PLoS Biol. 2026 Nov 3;24(11):e3003970. doi: 10.1371/journal.pbio.3003970 (PMC13730340; doi:10.1371/journal.pbio.3003970)
Supplement: Peer review history

# Editor report: Decision Letter 0

**DOI:** 10.1371/journal.pbio.3003970.r001

**Authors:** Ankiit Ahluwalia

27 Jan 2026

Dear Dr Smit,

Thank you for submitting your manuscript entitled "Adaptive fetal loss as a resolution of mother-offspring conflict in wild mandrills" for consideration as a Research Article by PLOS Biology.

Your manuscript has now been evaluated by the PLOS Biology editorial staff (as well as by an academic editor with relevant expertise) and I am writing to let you know that we would like to send your submission out for external peer review.

However, before we can send your manuscript to reviewers, we need you to complete your submission by providing the metadata that is required for full assessment. To this end, please login to Editorial Manager where you will find the paper in the 'Submissions Needing Revisions' folder on your homepage. Please click 'Revise Submission' from the Action Links and complete all additional questions in the submission questionnaire.

Once your full submission is complete, your paper will undergo a series of checks in preparation for peer review. After your manuscript has passed the checks it will be sent out for review. To provide the metadata for your submission, please Login to Editorial Manager (https://www.editorialmanager.com/pbiology) within two working days, i.e. by Jan 29 2026 11:59PM.

If your manuscript has been previously peer-reviewed at another journal, PLOS Biology is willing to work with those reviews in order to avoid re-starting the process. Submission of the previous reviews is entirely optional and our ability to use them effectively will depend on the willingness of the previous journal to confirm the content of the reports and share the reviewer identities. Please note that we reserve the right to invite additional reviewers if we consider that additional/independent reviewers are needed, although we aim to avoid this as far as possible. In our experience, working with previous reviews does save time.

If you would like us to consider previous reviewer reports, please edit your cover letter to let us know and include the name of the journal where the work was previously considered and the manuscript ID it was given. In addition, please upload a response to the reviews as a 'Prior Peer Review' file type, which should include the reports in full and a point-by-point reply detailing how you have or plan to address the reviewers' concerns.

During the process of completing your manuscript submission, you will be invited to opt-in to posting your pre-review manuscript as a bioRxiv preprint. Visit http://journals.plos.org/plosbiology/s/preprints for full details. If you consent to posting your current manuscript as a preprint, please upload a single Preprint PDF.

Feel free to email us at plosbiology@plos.org if you have any queries relating to your submission.

Kind regards,

Ankiit

Ankiit Ahluwalia,

PLOS Biology

aahluwalia@plos.org

# Peer review: Decision Letter 1

**DOI:** 10.1371/journal.pbio.3003970.r002

**Authors:** Ankiit Ahluwalia

23 Mar 2026

Dear Dr Smit,

Thank you for your patience while your manuscript "Adaptive fetal loss as a resolution of mother-offspring conflict in wild mandrills" was peer-reviewed at PLOS Biology. It has now been evaluated by the PLOS Biology editors, an Academic Editor with relevant expertise, and by several independent reviewers.

IMPORTANT: We think that your manuscript would be better considered as a Short Report. Your article has been changed to 'Short Reports' in our database.

In light of the reviews, which you will find at the end of this email, we would like to invite you to revise the work to thoroughly address the reviewers' reports.

As you will see below, the reviewers find the study potentially interesting and generally well done, however they also raise a number of concerns and they offer suggestions to strengthen the study further. We think the reviewer comments will need to be thoroughly addressed, including by providing additional reanalyses/or clarifying data to bolster the conclusions, reframing some of the findings, and discussing additional literature, before we can consider your study for publication.

After discussing these comments with the Academic Editor and the rest of the team, we have decided to invite a revision that should address all the points raised by the reviewers.

Given the extent of revision needed, we cannot make a decision about publication until we have seen the revised manuscript and your response to the reviewers' comments. Your revised manuscript is likely to be sent for further evaluation by all or a subset of the reviewers.

In addition to these revisions, you will need to complete some formatting changes, which you will receive in a follow up email. A member of our team will be in touch with a set of requests shortly.

We expect to receive your revised manuscript within 3 months. Please email us (plosbiology@plos.org) if you have any questions or concerns, or would like to request an extension.

At this stage, your manuscript remains formally under active consideration at our journal; please notify us by email if you do not intend to submit a revision so that we may withdraw it.

**IMPORTANT - SUBMITTING YOUR REVISION**

Your revisions should address the specific points made by each reviewer. Please submit the following files along with your revised manuscript:

1. A 'Response to Reviewers' file - this should detail your responses to the editorial requests, present a point-by-point response to all of the reviewers' comments, and indicate the changes made to the manuscript.

*NOTE: In your point-by-point response to the reviewers, please provide the full context of each review. Do not selectively quote paragraphs or sentences to reply to. The entire set of reviewer comments should be present in full and each specific point should be responded to individually, point by point.

You should also cite any additional relevant literature that has been published since the original submission and mention any additional citations in your response.

2. In addition to a clean copy of the manuscript, please also upload a 'track-changes' version of your manuscript that specifies the edits made. This should be uploaded as a "Revised Article with Changes Highlighted" file type.

*Re-submission Checklist*

When you are ready to resubmit your revised manuscript, please refer to this re-submission checklist: https://plos.io/Biology_Checklist

To submit a revised version of your manuscript, please go to https://www.editorialmanager.com/pbiology/ and log in as an Author. Click the link labelled 'Submissions Needing Revision' where you will find your submission record.

Please make sure to read the following important policies and guidelines while preparing your revision:

*Published Peer Review*

Please note while forming your response, if your article is accepted, you may have the opportunity to make the peer review history publicly available. The record will include editor decision letters (with reviews) and your responses to reviewer comments. If eligible, we will contact you to opt in or out. Please see here for more details:

https://blogs.plos.org/plos/2019/05/plos-journals-now-open-for-published-peer-review/

*PLOS Data Policy*

Please note that as a condition of publication PLOS' data policy (http://journals.plos.org/plosbiology/s/data-availability) requires that you make available all data used to draw the conclusions arrived at in your manuscript. If you have not already done so, you must include any data used in your manuscript either in appropriate repositories, within the body of the manuscript, or as supporting information (N.B. this includes any numerical values that were used to generate graphs, histograms etc.). For an example see here: http://www.plosbiology.org/article/info%3Adoi%2F10.1371%2Fjournal.pbio.1001908#s5

*Blot and Gel Data Policy*

We require the original, uncropped and minimally adjusted images supporting all blot and gel results reported in an article's figures or Supporting Information files. We will require these files before a manuscript can be accepted so please prepare them now, if you have not already uploaded them. Please carefully read our guidelines for how to prepare and upload this data: https://journals.plos.org/plosbiology/s/figures#loc-blot-and-gel-reporting-requirements

*Protocols deposition*

To enhance the reproducibility of your results, we recommend that if applicable you deposit your laboratory protocols in protocols.io, where a protocol can be assigned its own identifier (DOI) such that it can be cited independently in the future. Additionally, PLOS ONE offers an option for publishing peer-reviewed Lab Protocol articles, which describe protocols hosted on protocols.io. Read more information on sharing protocols at https://plos.org/protocols?utm_medium=editorial-email&utm_source=authorletters&utm_campaign=protocols

Thank you again for your submission to our journal. We hope that our editorial process has been constructive thus far, and we welcome your feedback at any time. Please don't hesitate to contact us if you have any questions or comments.

Sincerely,

Ankiit Ahluwalia,

PLOS Biology

aahluwalia@plos.org

------------------------------------

REVIEWS:

Reviewer #1: Thank you for the opportunity to read this exciting work! This was an interesting study in a system that permits a rare insight into these dynamics of fetal loss, without necessitating physiological sampling. As a short report, this provides a unique contribution to the literature with parallels to several (scant) works on the Bruce effect, and spontaneous fetal loss from other primates.

Generally, the article is clearly written and compelling. I have numerous comments mostly stemming from an interest in this work, or a perceived need for clarity. I do have a slight concern regarding the heaviness of the "adaptive" interpretation and fitness analyses and the mother-offspring conflict, which seem to be overly emphasized given the evidence here (as I understand it). While the statistical analyses are generally reasonable, there are a few nuances that I would benefit from clarity on.

There are other aspects of this system that I would be curious about: nutritional quality of the surrounding environment during fetal loss (NDVI, rainfall, or direct measures), female health during fetal loss (female condition, rank changes, etc.), male behavior during these periods (alpha male turnover, any tendencies towards infanticide?), rearing of the females themselves (captive versus wild reared; as rearing condition can alter maternal behavior). All of these could alter this system in ways that might influence fetal development, loss, and the general costs of reproduction. As the authors note for some of these, tantrums could be linked to maternal investment or condition. If the authors do not have some of these variables, then perhaps add a brief discussion of their absence as a limitation and future direction for alternative explanations.

Just an elaboration: though the article is focused on mother-offspring dynamics, the male behavior during these periods seems of direct relevance. This has relevance because infanticide has been reported in (captive) mandrills (Setchell et al. 2008) and my impression is that the Bruce effect has been invoked as a preventative for infanticidal strategies by new males (Roberts et al. 2012). The authors briefly explore coercion, but I have a few other questions: Was the alpha male stable across the study? Did fetal loss coincide with major changes in resident males? Even qualitative insights into these questions would be of interest to the target audience.

I am not wholly convinced that there is conflict here. The survival of the infant seems to be advantageous to both the mother and the infant. Lactation is, of course, the costlier process (Emery Thompson, 2024) and has already been invested into the living infant. Assuming mothers cannot support both offspring, then fetal loss is of mutual benefit for both mother and infant; yes? Though, the fetus has a conflicting priority at this stage - so, perhaps this is really inter-offspring conflict? Finally, there is a good deal of literature on the fetus' role in securing its own survival (e.g., Fowden & Moore, 2012; Sterner et al. 2012), which seems worth discussing and citing of the relevant works.

Finally, these rates of fetal loss seem well within the range of what we expect from all primates. If this is true, then this 'adaptive' fetal loss falls in the same range as 'general' (for lack of a better distinction) fetal loss. If this was adaptive for mandrills, why is the rate fairly consistent with what we see across primates? (see my comment to L168)

Section and Line Comments:

The title unambiguously defines fetal loss as adaptive, but the abstract presents resource allocation constraints as a (non-mutually exclusive) alternative. I would prefer adaptive removed from the title, if it is ambiguous throughout.

The Teaser is more appropriately couched in fitness outcomes (rather than an adaptive strategy)

Figure 1 is presented while the study is still being set up to the reader, yet is presenting study outcomes before they have been addressed in the text. I recognize this work is a short report, but this structural position was confusing.

Figure 2: If I understand correctly and all of these lines are plotted from the model posteriors, then add uncertainty to the plots. If they are not from model posteriors, then please re-plot them using model posteriors (and add uncertainty).

Figure 2c: I do not recall reading a rationale for the lower number of tantrums in infants of pregnant females? This would be beneficial to explicitly resolve or hypothesize about. Also, would it be better to know the x-axis relative to conception age or day until expected birth?

L126-132: During what period were the tantrum rates calculated? Note that I read through the relevant Methods section and this remained unclear to me.

Figure 3 & 143-161: Nuance is missing here in that females are unlikely to have only 2 offspring during their lifespan. It would be useful to know this all in the context of lifetime reproduction. The strategy of having multiple consecutive offspring seems more optimal in high quality circumstances, as supported by your findings of high ranking females practicing this strategy. If consecutive reproduction is successful, what is the benefit for lifetime reproductive output? If females are only having a lifetime output of 6-10 offspring, for instance, then the opportunity to have 3 infants in the span of 4 years (two consecutive, and one as females return to an 'every other season' cycle) is a big potential boon relative to a female sticking to the every-other-season strategy (which will yield 2 infants). The outcome would still be 2 infants were she to lose her middle infant, and still high (as you note in Figure 3) with fetal loss and rapid re-fertilization (3 infants). Some nuanced discussion of this is warranted given females have more than 2 offspring in their lives (I assume). More concisely, what are the lifetime fitness implications for females that: never have consecutive births, always have consecutive births with survival, or have a mixed strategy. Then present these same combinations but with fetal loss and infant death complications for lifetime fitness.

L168: The authors describe fetal loss as occurring in 10-20% of pregnancies across many primates. Given the observed fetal loss in mandrills is well within that range (52/356 * 100 = 14.6%), why do we need any explanatory variables here? That is, how does this circumstance differ from other primate systems? Why are these rates higher than we might expect from average rates attributable to random mutation or developmental complications? If they are not, then is there any reason to believe infants are not just cuing behavior off of the mother's behavior/physiology?

L174: I feel the need for some resolution, somewhere in the article, why there is a conflict? Assuming insufficient resources to support the fetus and lactation, then there is no conflict - because the sunk-cost in the living infant is of greater priority for the mother as well?

L189: I would value some elucidation of whether any of these fetal loss patterns were biased towards captive- or wild-reared mothers. I recognize that this would co-vary with age.

L281: Please describe "qualitatively similar". Did the meaningful effects remain?

Statistical Analyses

There is little mention of scaling variables (though I see, for instance, rank is standardized). Scaling is especially important when including binary and continuous variables (Gelman, 2007). Please confirm that continuous variables were scaled (and rerun analyses, if not, to confirm results are the same).

Please report outcomes of posterior predictive checks and screening for multicollinearity (pp_check() and pairs() plots in brms)

How do you discriminate between meaningful and non-meaningful effects? Are you relying on a 95% positive draw cut-off? Two-tailed or one-tailed?

Are your posterior draws based on the conditional or marginal posterior? Do these estimates and errors include variance from your random effects?

L325: Please cite R.

L461: Please cite Biorender in the captions of the relevant figures.

Supplementary

Several references are not populated or, rather, populated as: (?)

Please report correlation coefficients between Mother age, Infant age, and Mother rank. Also, did you screen for multicollinearity?

Please add, to all the statistical supplementary tables: lower/upper CIs and your meaningful cut-off for % Positive Draws. Also, add random effects and report Bayesian R2 estimates

Reviewer #2: The authors present an interesting study on fetal loss in wild mandrills. In this study, pregnancy and fetal loss were estimated by visually assessing the swellings of females. Using 11 years of data on 117 females (356 pregnancies), the authors tested what factors predict fetal loss and whether fetal loss is evolutionarily beneficial in certain contexts. The analyses suggest that pregnant females with a dependent infant face a higher risk of fetal loss than pregnant females without a dependent infant. Among pregnant females with dependent infants, higher rates of infant tantrums predicted a higher risk of fetal loss. Furthermore, the reproductive success of females who experience fetal loss was better than females who birthed an infant who then soon died. Together the authors posit that fetal loss is a strategy to maximize reproductive success in the context of a too-rapid reproductive pace. I am glad I said yes to this review because I enjoyed reading this report! I think the study will be a nice addition to the literature on female reproduction, maternal-offspring conflict, and life history.

There are a few aspects of the manuscript that could be clarified or improved.

First, infant presence vs recovery time.

"Infant presence, not limited recovery time, increases fetal loss" is the title of the first section. This may not be the most accurate way to frame the results. The analyses show that time since last gestation does not strongly predict fetal loss, but the presence of a dependent infant and time since presence of a dependent infant do strongly predict fetal loss. Since maternal care (lactation, carrying) is so costly, it seems odd to frame the findings this way. Recover time probably does matter; it's just that the mother has not even started recovery yet if there is still a dependent infant!

Similarly, there is a dichotomy presented at times, for example in the abstract: "fetal loss may reflect constraints on resource allocation or an adaptive adjustment to the high costs of short inter-birth intervals." While there are different perspectives, many would view fetal loss in response to resource constraints as a potentially adaptive response. If a pregnant female has limited resources, it might be most adaptive to terminate the pregnancy rather than invest the time and energy into a pregnancy that could lead to an infant that does not survive. Under the circumstances of resource constraints, this response would be adaptive. Perhaps some slight rewording could work: fetal loss may reflect an adaptive adjustment to constraints on resource allocation and/or the high costs of short IBIs.

Medium-term reproductive success.

The analysis to test proxies for fitness could be clarified a bit and/or improved. I understand the decision to use 'a period of two median interbirth intervals' as the unit of measurement, but I found it challenging to read and understand this throughout the paper. How many years is this? It might be easier on the reader to mention the time period instead (and justify the time period by saying it represents two median IBIs).

The window between first conception and next conception must vary across the 3 categories. Maybe this variation could be illustrated in Figure 3? Is the Bayesian t-test the best way to handle this analysis? The outcome variable seems to be a count (# of surviving infants in the time period), so would a Poisson be feasible? The main predictor of interest could be a categorical: fetal loss, next infant died, next infant survived. I also wonder if the authors considered using IBI as the outcome (similar to the cited Bruce effect paper Roberts et al). For an IBI analysis, the predictor categories of interest could be: two consecutive successful births, fetal loss between successful births/surviving infants, infant death between surviving infants.

Finally, I have a clarifying question about what the authors think is happening here! Is conceiving early just a mistake? Or do some females employ a fast reproductive strategy that works out often enough that it's adaptive on average (i.e., successful pregnancy and surviving infant sometimes) and mistakes can be somewhat mitigated by fetal loss if the pace is too fast at times? This is how I interpreted the conflict: mothers try to reject a lot when pregnant, but if the infant is exhibiting a lot of tantrums, then fetal loss may occur. So, this represents conflict and a negotiation. I do wonder if the tantrums are high and the mother does not experience fetal loss, what is the risk of infant death? (I'm not looking for the authors to provide an answer to this last question, just a thought for context and discussion)

Note: There are a number of "?"s in the supplementary materials

Reviewer #3: Smit et al. investigate the evolutionary and behavioral drivers of fetal loss in a wild population of mandrills. Using eleven years of data, including 356 pregnancies across 117 females, the authors conduct a strong empirical test the "adaptive fetal loss" hypothesis. They show that the presence of a dependent infant (under one year old) substantially increases the probability of fetal loss, and that females who experience fetal loss achieve higher medium-term reproductive success than those who carry an infant to term only for it to die shortly after birth.

I thoroughly enjoyed this interesting, well-written, and important manuscript and consider it the best paper I have reviewed in at least a year (for reference, I review about 25 manuscripts per year and edit roughly the same number). The study system (daily monitoring for 11 years, the ability to reliably identify cycling, pregnancy, and fetal loss) provides a lovely opportunity to test ideas central to life history theory and maternal-offspring conflict.

Analyses: I found the statistical analyses to be appropriate, clearly and succinctly explained, and transparently reported (e.g., Table S1-S10). The Bayesian GLMM framework allows for a nuanced understanding of the posterior probabilities of various drivers (reported as continuous values reflecting the percent positive posterior probability), rather than dichotomously characterizing effects as significant or non-significant.

Figures: Each of the three figures are effective, especially figure 1 that clearly depicts the conceptual timeline of female mandrill reproduction and figure 3, which is an outstanding visual depiction of the main results of the paper. I generally dislike the "graphical abstracts" sent out with email tables of contents from journals, in part because most of the figures are not that effective. If most graphical abstracts were as clear and effective as Smit et al.'s figure 3 I would feel differently. Very well done.

I see three areas where the authors could improve on the manuscript.

1. Overall, the authors were circumspect about the interpretation of their results and were admirably careful to not oversell their findings- with one exception. I found the causal language regarding the effect of mother-infant behavioral conflict to be too strong. For example, lines 18-19 stated that "behavioral conflict… triggers fetal loss", but this is supported by a correlation- there are no results (e.g., from non-invasively measured hormones) that causally link fetal loss through some stress-induced physiological pathway. I suggest the authors rein in their framing here.

2. There are unmeasured factors that could potentially confound the results. For example, while maternal rank and age are included as covariates, it is possible that maternal health could play a role here, too. Similarly, the random effect for year does not "control for" environmental confounds that might make years vary in quality. While the strength of the detected effects using the data presented reassure me that the results are likely robust, finding a way to assess the impacts of i) maternal health and ii) environmental quality (e.g., using phenology or rainfall data) would further strengthen the results and increase confidence in the conclusions.

3. As the authors transparently admit, pregnancy was detected via swelling, which occurs several weeks post-conception. This means the "true" rate of fetal loss is likely higher that they were able to detect. While this is an inherent limitation, I would have appreciated consideration of how this systematic undercounting might affect the results and interpretation.

Specific points

- Line 33: clarify who you mean by "their"

- Figure 2: would it be possible to include the Bayesian equivalent of confidence intervals, credibility intervals, on the lines in panels c-f?

- Line 167: the value 4.5% is not indicated anywhere in Figure 3. I suggest in these parenthesis the authors indicate that 4.5% is 8 of 176, to tie it more clearly to the figure.

- When models included random effects the authors noted them as "random factors" (e.g., 261, 294, 311). Please specify what the random effects are. I assume they are random intercepts, but it is possible they could be random slopes, random slopes and intercepts (correlated or uncorrelated), so specifying would be welcome.

# Author comment: Author response to Decision Letter 2

**DOI:** 10.1371/journal.pbio.3003970.r003

29 May 2026

Attachment Submitted filename: ResponsesToReviewFetalLoss_v37.pdf

# Peer review: Decision Letter 2

**DOI:** 10.1371/journal.pbio.3003970.r004

**Authors:** Ankiit Ahluwalia

13 Jul 2026

Dear Dr Smit,

Thank you for your patience while we considered your revised manuscript "Infant presence and behavior predict maternal fetal loss in wild mandrills" for publication as a Short Reports at PLOS Biology. This revised version of your manuscript has been evaluated by the PLOS Biology editors, the Academic Editor and the two original reviewers (Reviewer1 and Reviewer2 in the first round).

Based on the reviews , we are likely to accept this manuscript for publication, provided you satisfactorily address the remaining points raised by the reviewers. Please also make sure to address the following data and other policy-related requests.

IMPORTANT - please attend to the following:

a) Overall, the Academic Editor is largely satisfied and has provided some comments for your consideration (below).

b) Please address my Data Policy requests below; specifically, you need to supply the underlying data for Figs 2ABCD, 3, 4, S1, S2. The data needs associated data repository, such as Zenodo with a DOI or a supplementary data file.

c) Please clearly cite the location of the data in all relevant main and supplementary figure legends, for example: “The data underlying this figure can be found at doi.org/10.xxx/zenodo.xxxxx.” We understand that this may appear repetitive, but it helps make the figures and their legends more self contained.

d) Please include the URLs of your funders in the Financial Disclosure statement.

e) Please make any custom code available, either as a supplementary file or as part of your data deposition.

f) In order to make the Title more specific and appealing, please change it to “Infant presence and tantrum behavior predict maternal fetal loss in wild mandrills”.

**AE Comments (Lightly Edited)**

I have read Reviewer 1’s comments and I agree with overall assessment. The authors should be able to deal with most of these comments easily and take up the reviewer’s suggestions. For the few comments where the authors might disagree they should be able to include a short explanation in the text as to why they took the route they did (e.g., justify the statistical approach they took).

As you address these items, please take this last chance to review your reference list to ensure that it is complete and correct. If you have cited papers that have been retracted, please include the rationale for doing so in the manuscript text, or remove these references and replace them with relevant current references. Any changes to the reference list should be mentioned in the cover letter that accompanies your revised manuscript.

In addition to these revisions, you may need to complete some formatting changes, which you will receive in a follow up email. A member of our team will be in touch with a set of requests shortly. If you do not receive a separate email within a few days, please assume that checks have been completed, and no additional changes are required.

We expect to receive your revised manuscript within three weeks.

To submit your revision, please go to https://www.editorialmanager.com/pbiology/ and log in as an Author. Click the link labelled 'Submissions Needing Revision' to find your submission record. Your revised submission must include the following:

- a cover letter that should detail your responses to any editorial requests, if applicable, and whether changes have been made to the reference list

- a Response to Reviewers file that provides a detailed response to the reviewers' comments (if applicable, if not applicable please do not delete your existing 'Response to Reviewers' file.)

- a track-changes file indicating any changes that you have made to the manuscript.

NOTE: If Supporting Information files are included with your article, note that these are not copyedited and will be published as they are submitted. Please ensure that these files are legible and of high quality (at least 300 dpi) in an easily accessible file format. For this reason, please be aware that any references listed in an SI file will not be indexed. For more information, see our Supporting Information guidelines:

https://journals.plos.org/plosbiology/s/supporting-information

*Published Peer Review History*

Please note that you may have the opportunity to make the peer review history publicly available. The record will include editor decision letters (with reviews) and your responses to reviewer comments. If eligible, we will contact you to opt in or out. Please see here for more details:

https://plos.org/published-peer-review-history/

*Press*

Should you, your institution's press office or the journal office choose to press release your paper, please ensure you have opted out of Early Article Posting on the submission form. We ask that you notify us as soon as possible if you or your institution is planning to press release the article.

*Protocols deposition*

To enhance the reproducibility of your results, we recommend that if applicable you deposit your laboratory protocols in protocols.io, where a protocol can be assigned its own identifier (DOI) such that it can be cited independently in the future. Additionally, PLOS ONE offers an option for publishing peer-reviewed Lab Protocol articles, which describe protocols hosted on protocols.io. Read more information on sharing protocols at https://plos.org/protocols?utm_medium=editorial-email&utm_source=authorletters&utm_campaign=protocols

Please do not hesitate to contact me should you have any questions.

Sincerely,

Ankiit

Ankiit Ahluwalia,

aahluwalia@plos.org

PLOS Biology

------------------------------------------------------------------------

****[DELETE AS APPROPRIATE]****

ETHICS STATEMENT:

-- Please include the full name of the IACUC/ethics committee that reviewed and approved the animal care and use protocol/permit/project license. Please also include an approval number.

-- Please include the specific national or international regulations/guidelines to which your animal care and use protocol adhered. Please note that institutional or accreditation organization guidelines (such as AAALAC) do not meet this requirement.

-- Please include information about the form of consent (written/oral) given for research involving human participants. All research involving human participants must have been approved by the authors' Institutional Review Board (IRB) or an equivalent committee, and must have been conducted according to the principles expressed in the Declaration of Helsinki.

------------------------------------------------------------------------

DATA POLICY:

You may be aware of the PLOS Data Policy, which requires that all data be made available without restriction: http://journals.plos.org/plosbiology/s/data-availability. For more information, please also see this editorial: http://dx.doi.org/10.1371/journal.pbio.1001797

Note that we do not require all raw data. Rather, we ask that all individual quantitative observations that underlie the data summarized in the figures and results of your paper be made available in one of the following forms:

1) Supplementary files (e.g., excel). Please ensure that all data files are uploaded as 'Supporting Information' and are invariably referred to (in the manuscript, figure legends, and the Description field when uploading your files) using the following format verbatim: S1 Data, S2 Data, etc. Multiple panels of a single or even several figures can be included as multiple sheets in one excel file that is saved using exactly the following convention: S1_Data.xlsx (using an underscore).

2) Deposition in a publicly available repository. Please also provide the accession code or a reviewer link so that we may view your data before publication.

Regardless of the method selected, please ensure that you provide the individual numerical values that underlie the summary data displayed in the following figure panels as they are essential for readers to assess your analysis and to reproduce it:

[Figs….]

NOTE: the numerical data provided should include all replicates AND the way in which the plotted mean and errors were derived (it should not present only the mean/average values).

Please also ensure that figure legends in your manuscript include information on where the underlying data can be found, and ensure your supplemental data file/s has a legend.

Please ensure that your Data Statement in the submission system accurately describes where your data can be found.

------------------------------------------------------------------------

APPROPRIATE DATA PRESENTATION?

(If you think the paper is not using best practice, add the following)

- Please ensure that you are using best practice for statistical reporting and data presentation. These are our guidelines https://journals.plos.org/plosbiology/s/best-practices-in-research-reporting#loc-statistical-reporting and a useful resource on data presentation https://journals.plos.org/plosbiology/article?id=10.1371/journal.pbio.1002128

- If you are reporting experiments where n ≤ 5, please plot each individual data point.

------------------------------------------------------------------------

CODE POLICY

Per journal policy, if you have generated any custom code during the course of this investigation, please make it available without restrictions. Please ensure that the code is sufficiently well documented and reusable, and that your Data Statement in the Editorial Manager submission system accurately describes where your code can be found. More information on our Code Policy, what and how to share can be found here: https://journals.plos.org/plosbiology/s/code-availability

Please note that we cannot accept sole deposition of code in GitHub, as this could be changed after publication. However, you can archive this version of your publicly available GitHub code to Zenodo. Once you do this, it will generate a DOI number, which you will need to provide in the Data Accessibility Statement (you are welcome to also provide the GitHub access information). See the process for doing this here: https://docs.github.com/en/repositories/archiving-a-github-repository/referencing-and-citing-content

------------------------------------------------------------------------

SPECIES INDICATED IN THE ABSTRACT?

- Please note that per journal policy, the model system/species studied should be clearly stated in the abstract of your manuscript.

**EDITOR, CONSIDER ALSO INCLUDING ORGANISM IN THE TITLE, IF SENSICAL/RELEVANT AND/OR TO AVOID BEING MISLEADING/OVERREACHING (not always preferred)**

------------------------------------------------------------------------

BLOT AND GEL REPORTING REQUIREMENTS:

We require the original, uncropped and minimally adjusted images supporting all blot and gel results reported in an article's figures or Supporting Information files. We will require these files before a manuscript can be accepted so please prepare and upload them now. Please carefully read our guidelines for how to prepare and upload this data: https://journals.plos.org/plosbiology/s/figures#loc-blot-and-gel-reporting-requirements

------------------------------------------------------------------------

DATA NOT SHOWN?

- Please note that per journal policy, we do not allow the mention of "data not shown", "personal communication", "manuscript in preparation" or other references to data that is not publicly available or contained within this manuscript. Please either remove mention of these data or provide figures presenting the results and the data underlying the figure(s).

------------------------------------------------------------------------

Reviewer remarks:

Reviewer #1: I want to emphasize my appreciation for the authors taking into consideration my comments from the last review. I recognize that re-running models with new variables can be time consuming. The majority of my questions and comments have been addressed. I do have a few more comments and suggestions, mostly in the presentation of results.

As an aside, I would encourage the authors to generate a compare document with track changes for reviewers in the future. This can be accomplished in many ways that should be compatible with a Latex to doc workflow. Three options come to mind: Overleaf offers a compare function, the authors can compare *.doc files using Word or LibreOffice Writer (which is free), or latexdiff might work. Additionally, please include line numbers for where changes occur (which were only included sometimes). Both of these changes improve the expedience and ease of the review process.

I understand the desire to not rely on CIs. Even so, it is still essential to include a statement about how the authors interpret their estimates, posterior draws, and effect sizes. Then be consistent in decision making and terminology. For example, in their response the authors state they "have now used expressions like 'weakly correlated' while always reporting posterior probabilities." How do the authors decide what is 'weak' (e.g., L63, L75, L118)? This comment is also applicable to other phrases, like: "little association" (L131), &: "this difference is not statistically supported (50% posterior probability)" (L184). And similarly throughout, where applicable. Do these descriptors differ from each other? If so please unify them, in addition to providing clear text for how you made your inferences. This is essential for readers to understand the authors' rationale.

In the response, the authors state that: "…our posterior draws are based on the conditional posteriors (which are also more interpretable). These estimates and standard errors do not include variance from the random effects". It is my understanding that these are not Conditional effects. I believe the authors use as_draws_df() which, I believe, provides Marginal effects. My understanding is that Marginal effects do not include variance from REs, while Conditional effects do. Regardless, I think it would be best practice to just report both types of posterior draws. Consider using conditional effects for a typical hypothetical group. E.g., something like: posterior_epred(model, … , re_formula = NULL, allow_new_levels = TRUE, sample_new_levels = "uncertainty"). And marginal effects, something like: posterior_epred(model, … , re_formula = NA). Conditional effects are worthwhile to understand whether the findings are robust relative to group-level variance from this population (not just generalizability).

Maternal rank seemed to be a recurring variable of interest (5/6 models had many [>90%] negative draws - assuming no draws were exactly zero), yet I did not find much discussion of this variable. Indeed there is no mention of [maternal] rank in the Results/Discussion between lines 42 (Introduction) to 284 (Methods). Given its influence in the models, please add this content to the discussion.

Line-by-line comments:

L64: The end of this line is cut-off

L122: "Rejection probability in pregnant and non-pregnant females converge…" should be "Rejection probabilities…", yes?

L122: How do you qualify/quantify convergence?

L134: Figure 2c should be Figure 2d, yes?

L145-169: This paragraph had some odd transitions. The work starts with female-female competition, only really discusses male takeovers. Then, it feels like the new sentence on L163 starts a different paragraph. Please revise this section for clarity. My recommendation would be that L144-163 would work better as its own subsection.

L153: The authors bring up female-female competition, but it is unclear what variables assesses that. I might assume that it is maternal rank? If so, then that finding can be intuitively discussed here. Instead, only male takeovers is discussed. My supposition is that both are important, but I would value the authors' explicit insights.

L193-194: Please add precision as to what timeframes you mean when stating: "short-term" and "medium-term"?

L204: I think a Conclusion subheading would make the transition from the preceding text less awkward.

L204-222: Adding your alpha male takeovers finding might connect this work more completely with relevant literature.

L310-329: I found the presentation of rho with the posterior draws confusing given only the Bayesian GLMM has been discussed here. Traditionally and in the rest of your manuscript, the estimate is presented with posterior draws. I suggest making the Spearman's correlations distinct from the posterior draws, or clarify what is going on in this passage if I have misinterpreted.

L412: What was the number of warm-up iterations and the value for thin?

R & its packages: Please add version numbers for the version of R you used, and the packages. Or, confirm that these details are clear in your code/readmes.

The Supplementary Materials still has content populated as: [?]. Especially in the Supplementary Analysis on Sexual Coercion.

Reviewer #2: I am pleased with this revised manuscript. The authors responded to each of my questions and suggestions in an acceptable manner. I think the authors have produced an interesting and valuable piece of work.

Attachment Submitted filename: PBIOLOGY-D-26-00056_R2_Review - Final.docx

# Author comment: Author response to Decision Letter 3

**DOI:** 10.1371/journal.pbio.3003970.r005

25 Jul 2026

Attachment Submitted filename: ResponsesToReviewFetalLoss_v39.pdf

# Editor report: Decision Letter 3

**DOI:** 10.1371/journal.pbio.3003970.r006

**Authors:** Ankiit Ahluwalia

12 Aug 2026

Dear Dr Smit,

Thank you for the submission of your revised Short Reports "Infant presence and mother-infant conflict predict fetal loss in wild mandrills" for publication in PLOS Biology. On behalf of my colleagues and the Academic Editor, Michael Jennions, I am pleased to say that we can in principle accept your manuscript for publication, provided you address any remaining formatting and reporting issues. These will be detailed in an email you should receive within 2-3 business days from our colleagues in the journal operations team; no action is required from you until then. Please note that we will not be able to formally accept your manuscript and schedule it for publication until you have completed any requested changes.

Please take a minute to log into Editorial Manager at http://www.editorialmanager.com/pbiology/, click the "Update My Information" link at the top of the page, and update your user information to ensure an efficient production process.

PRESS

We frequently collaborate with press offices. If your institution or institutions have a press office, please notify them about your upcoming paper at this point, to enable them to help maximise its impact. If the press office is planning to promote your findings, we would be grateful if they could coordinate with biologypress@plos.org. If you have previously opted in to the early version process, we ask that you notify us immediately of any press plans so that we may opt out on your behalf.

We also ask that you take this opportunity to read our Embargo Policy regarding the discussion, promotion and media coverage of work that is yet to be published by PLOS. As your manuscript is not yet published, it is bound by the conditions of our Embargo Policy. Please be aware that this policy is in place both to ensure that any press coverage of your article is fully substantiated and to provide a direct link between such coverage and the published work. For full details of our Embargo Policy, please visit http://www.plos.org/about/media-inquiries/embargo-policy/.

Thank you again for choosing PLOS Biology for publication and supporting Open Access publishing. We look forward to publishing your study.

Sincerely,

Ankiit Ahluwalia,

PLOS Biology

aahluwalia@plos.org
